# Supplementary material for: Blue-to-Red TagFT, mTagFT, mTsFT, and Green-to-FarRed mNeptusFT2 Proteins, Genetically Encoded True and Tandem Fluorescent Timers
Source: Int J Mol Sci. 2023 Feb 7;24(4):3279. doi: 10.3390/ijms24043279 (PMC9963904; doi:10.3390/ijms24043279)
Supplement: Supplementary file 1 [file ijms-24-03279-s001.zip › TagFT_Supplementary information_Proofreaded.pdf]

Supplementary Information

The Blue-to-Red TagFT, mTagFT, mTsFT, and Green-to-FarRed mNeptusFT2 Proteins, Genetically Encoded True and Tandem Fluorescent Timers

Oksana M. Subach, Anna V. Vlaskina, Yulia K. Agapova, Alena Y. Nikolaeva, Konstantin V. Anokhin, Kiryl D. Piatkevich, Maxim V. Patrushev, Konstantin M. Boyko and Fedor V. Subach

Supplementary Tables and Figures

**Table S1**	**2**
**Table S2**	**6**
**Figure S1**	**8**
**Figure S2**	**11**
**Figure S3**	**12**
**Figure S4**	**13**
**Figure S5**	**14**
**Figure S6**	**15**
**Figure S7**	**17**
**Figure S8**	**18**
**Figure S9**	**19**
**Figure S10**	**20**
**Figure S11**	**21**
**Figure S12**	**22**
**Figure S13**	**23**
**Figure S14**	**24**
**Figure S15**	**25**
**Video S1**	**26**
**Supplementary Methods**	**27**
**Supplementary references**	**32**

Table S1. List of primers.

Primer	Primer sequence (5′-3′)	Comment
**Fw-BglII-(PA)TagRFP**	GCTCGAGATCTATGGTGTCTAAGGGCGAAGAG	Cloning of TagFT and mTagFT into pBAD/HisB vector at BglII/EcoRI sites.
**Rv-EcoRI-mKate2(PATagRFP)**	GCTCGGAATTCCTATCTGTGCCCCAGTTTGCTAG	
**Fw-BglII-TagBFP**	GCTCGAGATCTATGAGCGAGCTGATTAAGGAG;	Cloning of mTsFT into pBAD/HisB vector at BglII/HindIII sites.
**mCherry-HindIII-r**	GATAAGCTTTTACTTATACAG CTCGTC	
**Fw-BglII-TagBFP**	GCTCGAGATCTATGAGCGAGCTGATTAAGGAG;	Cloning of mNeptusFT into pBAD/HisB vector at BglII/HindIII sites.
**Neptune-HindIII-r**	cgtAAGCTTttacttgtacagctcgtccatgcc	
**Fw-BglII-TagBFP**	GCTCGAGATCTATGAGCGAGCTGATTAAGGAG;	Random mutagenesis of mTagBFP part in mTsFT timer with cloning at BglII/EcoRI sites
**BFP-EcoRI-r**	CATgaattcattgagcttgtgccccagtttG	
**Fw-BglII-TagBFP**	GCTCGAGATCTATGAGCGAGCTGATTAAGGAG;	Random mutagenesis of mNeptune part in mNeptusFT timer with cloning at BglII/EcoRI sites
**Nep-EcoRI-r**	GTC GAA TTC ATT GAG CTT ATG GCC GAG	
**TagFT-NheI2**	TCC GCT AGC GGT ACC GGT CGC CAC CAT GGT GTC TAA GGG CGA AGA G	Cloning of the TagFT and mTagFT timers into pAAV-CAG-iRFP-P2A-EGFP plasmid.
**TagFT-AgeI-r**	TGA ACC GGT CGT CTG TGC CCC AGT TTG CTA G	
**Neptune-KpnI**	CCC GGT ACC GCC ACC ATG AGC GAG CTG ATT AAG	Cloning of the mTsFT timer into pAAV-CAG-iRFP-P2A-EGFP plasmid.
**RubyFT-AgeI-r**	TGA ACC GGT CGC TTG TAC AGC TCG TCC ATG	
**BamHI-mTFT**	ccgggatccaccggtcgccaccATGGTGTCTAAGGGCGAAGAG	Cloning of the mTagFT and TagFT timers into pLU-vimentin-NeonOxIrr plasmid.
**mTFT-XbaI-r**	actTCTAGAttaTCTGTGCCCCAGTTTGCTAG	
**BamHI-Neptune**	ccgggatccaccggtcgccaccATGAGCGAGCTGATTAAG	Cloning of the mTsFT and mNeptusFT2 timers into pLU-vimentin-mTagFT plasmid.
**LSSmSc-XbaI-r**	actTCTAGAttaCTTGTACAGCTCGTCCATGcc	
**TagFT-NheI2**	TCC GCT AGC GGT ACC GGT CGC CAC CAT GGT GTC TAA GGG CGA AGA G	Cloning of the mTagFT timer into pTagBFP-actin and pTagGFP2-tubulin plasmids.
**TagFT-BglII-r**	TCG AGA TCT CCT AGG GAG TCC GGA TCT GTG CCC CAG TTT GCT AG	
**mTagFT-83**	CAC CCA GGG CAT CCC CGA CTD KTD KAA GCA GTC CTT CCC TGA GG	Generation of overlap library of TagRFP protein at indicated positions.
**mTagFT-83-r**	CCT CAG GGA AGG ACT GCT TMH AMH AGT CGG GGA TGC CCT GGG TG	
**mTagFT-148-r**	CAG GCC GCC GTC AGC GGG CTT CAG TTT CTC GGT GYT GGC CTC CCA GCC GAG TG	
**mTagFT-165**	CCC GCT GAC GGC GGC CTG GAA GGC AGA NNS GAC ATG GCC CTG AAG CTC	
**mTagFT-165-r**	GAG CTT CAG GGC CAT GTC SNN TCT GCC TTC CAG GCC GCC GTC AGC GGG	
**mTagFT-181**	GGC CAC CTG ATC TGC AAC YTT AAG ACC ACA TAC AGA TCC	
**mTagFT-181-r**	GGA TCT GTA TGT GGT CTT AAR GTT GCA GAT CAG GTG GCC	
**mTagFT-220-r**	GTG CCC CAG TTT GCT AGG GAG GTC CGA GTA TCT GGC CAC ASH CAC CTC GTG CWG CTC GAC GTA GGT CTC TTT G	
**HypKat-148X**	cgatcaggcaatgtccgccggcNNSaccgagaccctgtaccccgctg	
**HypKat-148X-r**	cagcggggtacagggtctcggtSNNgccggcggacattgcctgatcg	
**HypKat-147**	gaaaacactcggctgggaggcc ggcaccggtttctgttttgaagc	

**HypKat-147-r**	gcttcaaaacagaaaccggtgccggcctcccagccgagtgttttc	
**HypKat-144**	gatgcagaagaaaacactcggc ggcaccggtttctgttttgaagc;	
**HypKat-144-r**	gcttcaaaacagaaaccggtgccgccgagtgttttcttctgcatc	
**HypBFP-145X**	cgatcaggcaatgtccgccggcNNSgaggccttcaccgagacgctgtac	
**HypBFP-145X-r**	gtacagcgtctcggtgaaggcctcSNNgccggcggacattgcctgatcg	
**HypKat-145X**	cgatcaggcaatgtccgccggcNNSgaggcctccaccgagaccctgtac	
**HypKat-145X-r**	gtacagggtctcggtggaggcctcSNNgccggcggacattgcctgatcg	
**Fw-BglII-18MVL**	GAC AGA TCT ATG GTG AGC GAG CTG ATT AAG GAG AAC ATG CAC ATG AAG CTG TAC VTG GAG GGC ACC GTG AAC AAC CAC C	
**Rv-69KRH**	GTC GGG GAT GCC CTG GGT GTG GTT GAT GAA GGT KYK GCT GCC GTA CAT GAA GCT GG	
**Fw-84WF**	CAC ACC CAG GGC ATC CCC GAC TTC TKK AAG CAG TCC TTC CCY GAG GG	
**Rv-152MIL-TagRFP**	GCT TCA GGG CCA TGT CGC TTC TGC CTT CCA GGC CGC CGT CAG CGG GGT ASA KCA TCT CGG TGT TGG CCT C	
**Rv-152MIL-mKate**	GCT TCA GGG CCA TGT CGG CTC TGC CTT CCA GGC CGC CGT CAG CGG GGT ASA KGG TCT CGG TGG AGG CCT C	
**Fw-179VAC**	CGA CAT GGC CCT GAA GCT CGT GGG CGG GGG CCA CCT GAT CKB CAA CTT SAA GAC CAC ATA CAG	
**Rv-203,205KRHML**	GGC CTC CTT GAT TCT TTC CAK TCT KYK GTC CAC ATA GTA GAC GCC	
**Fw-203,205KRHML**	GGC GTC TAC TAT GTG GAC MRM AGA MTG GAA AGA ATC AAG GAG GCC	
**Rv-EcoRI-224SA**	GAC GAA TTC ATT AAG TTT GTG CCC CAG TTT GCT AGG GAG GTC GCA GTA TCT GGC CAC AGM CAC CTC GTG CTG CTC GAC	
**Fw-16**	GGAGAACATGCACATGAAGNNSNNSNNSACCGGTTTCTGTT TTGAAGCCGG	
**Rv-16**	CCGGCTTCAAAACAGAAACCGGTSNNSNNSNNCT TCATGTGCATGTTCTCC	
**Fw-17**	GTTTGCGCGATCAGGCAATGTCCNNSNNSNNSATGGAGGG CACCGTGRACAAC	
**Rv-17**	GTTGTYCACGGTGCCCTCCATSNNSNNSNNGGACATTGCCT GATCGCGCAAAC	
**Fw-44**	GTTTGCGCGATCAGGCAATGTCCNNSNNSNNSAGAATCAA GGYGGTCGAGGG	
**Rv-44**	CCCTCGACCRCCTTGATTCTSNNSNNSNNGGACATTGCCTG ATCGCGCAAAC	
**Fw-43**	GCCCTACGAGGGCACCCAGNNSNNSNNSACCGGTTTCTGTT TTGAAGCCGG	
**Rv-43**	CCGGCTTCAAAACAGAAACCGGTSNNSNNSNNCTGGGTGC CCTCGTAGGGC	
**Fw-123**	GTTTGCGCGATCAGGCAATGTCCNNSNNSNNSAAGATCAG AGGGGTGAACTTC	
**Rv-123**	GAAGTTCACCCCTCTGATCTTSNNSNNSNNGGACATTGCCT GATCGCGCAAAC	
**Fw-122**	CAGGACGGCTGCCTCATCTACNNSNNSNNSACCGGTTTCTG TTTTGAAGCCGG	
**Rv-122**	CCGGCTTCAAAACAGAAACCGGTSNNSNNSNNGTAGATGA GGCAGCCGTCCTG	
**OxKat203**	gtttgcgcgatcaggcaatgtccNNSNNSNNSagactggaaagaatcaaggagg	
**OxKat203-r**	cctCCTTGATTCTTTCCAGTCTSNNSNNSNNGGACATTGCCTG ATCGCGCAAAC	

**KatOx203**	gatgccYggcgtctactatgtgNNSNNSNNSaccggtttctgttttgaagcc	
**KatOx203-r**	GGCTTCAAAACAGAAACCGGTSNNSNNSNNCACATAGTAG ACGCCRGGCATC	
**OxKat206**	gtttgcgcgatcaggcaatgtccNNSNNSNNSagaatcaaggaggccRac	
**OxKat206-r**	GTYGGCCTCCTTGATTCTSNNSNNSNNGGACATTGCCTGATC GCGCAAAC	
**BFPOx-206**	ctactatgtggactacagaNNSNNSNNSaccggtttctgttttgaagcc	
**BFPOx-206-r**	GGCTTCAAAACAGAAACCGGTSNNSNNSNNTCTGTAGTCC ACATAGTAG	
**OxKat182**	gtttgcgcgatcaggcaatgtccNNSNNSNNSaccacataYagatccaagaaacc	
**OxKat182-r**	GGTTTCTTGGATCTRTATGTGGTSNNSNNSNNGGACATTGCC TGATCGCGCAAAC	
**KatOx182**	gggccacctgatctgcaacNNSNNSNNSaccggtttctgttttgaagcc	
**KatOx182-r**	GGCTTCAAAACAGAAACCGGTSNNSNNSNNGTTGCAGATC AGGTGGCCC	
**BFPOx182**	gagccatctgatcgcaaacNNSNNSNNSaccggtttctgttttgaagcc	
**BFPOx182-r**	GGCTTCAAAACAGAAACCGGTSNNSNNSNNGTTTGCGATC AGATGGCTC	
**OxKat8-205**	GTTTGCGCGATCAGGCAATGNNSNNSNNSCCGGAAAGAAT CAAGGAGGC	
**OxKat8-205-rv**	GCCTCCTTGATTCTTTCCGGSNNSNNSNNCATTGCCTGATCG CGCAAAC	
**Kat8Ox-205**	GTCTACTATGTGGACAACTTGNNSNNSNNSGGTTTCTGTTTT GAAGCCGG	
**Kat8Ox-205-rv**	CCGGCTTCAAAACAGAAACCSNNSNNSNNCAAGT TGTCCACATAGTAGAC	
**mTFT-L16E**	GAGATCATGCACATTAAGgaGTACATGGAGGGCACCGTG	Directed mutagenesis of the mTagFT timer.
**mTFT-L16E-r**	CACGGTGCCCTCCATGTACtcCTTAATGTGCATGATCTC	
**mTFT-M44C**	CTACGAGGGCACCCAGACCtgcAGAGTCAAGGTGGTCGAG	
**mTFT-M44C-r**	CTCGACCACCTTGACTCTgcaGGTCTGGGTGCCCTCGTAG	
**mTFT-M44Q**	CTACGAGGGCACCCAGACCcaGAGAGTCAAGGTGGTCGAG	
**mTFT-M44Q-r**	CTCGACCACCTTGACTCTCtgGGTCTGGGTGCCCTCGTAG	
**mTFT-L65M**	CATCCTGGCTACCAGCTTCaTGTACGGCAGCAGGACCTTTAT C	
**mTFT-L65M-r**	GATAAAGGTCCTGCTGCCGTACAtGAAGCTGGTAGCCAGGA TG	
**mTFT-S148I**	CACTCGGCTGGGAGGCCAtCACCGAGAAACTGAAGCCC	
**mTFT-S148I-r**	GGGCTTCAGTTTCTCGGTGaTGGCCTCCCAGCCGAGTG	
**mTFT-S165A**	GGCGGCCTGGAAGGCAGAgcgGACATGGCCCTGAAGCTC	
**mTFT-S165A-r**	GAGCTTCAGGGCCATGTCcgcTCTGCCTTCCAGGCCGCC	
**mTFT-H203I**	GGCGTCTACTATGTGGACatCAGACTGGAAAGAATCAAG	
**mTFT-H203I-r**	CTTGATTCTTTCCAGTCTGatGTCCACATAGTAGACGCC	
**mTFT-L220Q**	CAAAGAGACCTACGTCGAGCaGCACGAGGTGTCTGTAGCC	
**mTFT-L220Q-r**	GGCTACAGACACCTCGTGCtGCTCGACGTAGGTCTCTTTG	
**mTFT-S224A**	GTCGAGCTGCACGAGGTGgCTGTAGCCAGATACCCGGAC	
**mTFT-S224A-r**	GTCCGGGTATCTGGCTACAGcCACCTCGTGCAGCTCGAC	

**TFTN-NheI**	AccGCTAGCATGGTGTCTAAGGGCGAAG	Cloning of split versions of FTs into pAAV-CAG-bJun/bFos/bFosΔZip plasmids
**MFTN-NheI**	AccGCTAGCATGGTAAGCAAGGGCGAG	
**TFTN-EcoRI-r**	cttgaattcctaGTCAGCGGGGTACAGCAC	
**mTFTN-EcoRI-r**	cttgaattcctaGTCAGCGGGCTTCAGTTTC	
**mRFTN-EcoRI-r**	cttgaattcctaATCTGCTGGATACATCATC	
**MFTN-EcoRI-r**	cttgaattcctaGTCCTCGGGGTATATCCG	
**MFTN-HindIII-r**	cttAAGCTTctaGTCCTCGGGGTATATCCG	
**TFTC-NheI**	AccGCTAGCGGCGGCCTGGAAGGCAGAG	
**mTFTC-NheI**	AccGCTAGCGGCGGCCTGGAAGGCAGAAG	
**mRFTC-NheI**	AccGCTAGCGGTGGTCTGAGGGGATAC	
**MFTC-NheI**	AccGCTAGCGGCGCCCTGAAGGGCGAG	
**Neptune-KpnI**	CCC GGT ACC GCC ACC ATG AGC GAG CTG ATT AAG	Cloning of mNeptusFT1/2 into pSBbi-GN-iRFP-hCdt1(1-100)-Puromycin vector
**hGem-HindIII-r**	GCC AAG CTT TTA CAG CGC CTT TCT CCG TTT TTC	
**Neptune-KpnI**	CCC GGT ACC GCC ACC ATG AGC GAG CTG ATT AAG	Cloning of mTsFT into pSBbi-GN-iRFP-hCdt1(1-100)-Hygromycin vector
**hCdt1-HindIII-r**	GCC AAG CTT TTA TTT CTT TAT CTT CTG GCC CG	
**FastFT-KpnI**	CCC GGT ACC GCC ACC ATG GTG AGC AAG GGC GAG	Cloning of MediumFT, FastFT, and FastFT2 into pSBbi-GN-iRFP-hCdt1(1-100)-Hygromycin vector
**hCdt1-HindIII-r**	GCC AAG CTT TTA TTT CTT TAT CTT CTG GCC CG	
**TagFT-KpnI**	CCC GGT ACC GCC ACC ATG GTG TCT AAG GGC GAA GAG	Cloning of TagFT and mTagFT into pSBbi-GN-iRFP-hCdt1(1-100)-Hygromycin vector
**hCdt1-HindIII-r**	GCC AAG CTT TTA TTT CTT TAT CTT CTG GCC CG	

Table S2. Data collection, processing, and refinement.

**Data Collection**	
Diffraction source	BL41XU, SPring8
Wavelength (Å)	1.0
Temperature (K)	100
Detector	EIGER
Crystal-to-detector distance (mm)	465.00
Rotation range per image (°)	0.5
Total rotation range (°)	160
Space group	P2
a, b, c (Å)	60.65, 95.32, 95.38
α, β, γ (°)	90.0; 90.0; 90.0
Unique reflections	23481 (3823)
Resolution range (Å)	95.38 –2.90 (3.08–2.90)
Completeness (%)	97.3 (98.2)
Average redundancy	2.6 (2.25
$\langle I/\sigma(I)\rangle$	4.7 (1.1)
Rpim (%)	12.3 (94.5)
$CC_{1/2}$	97.4 (33.7)
**Refinement**	
R_{fact} (%)	24.2
$R_{free.}$ (%)	27.7
Bonds (Å)	0.01
Angles (°)	1.08
**Ramachandran plot**	
Most favored (%)	92.9
Allowed (%)	4.8
**No. atoms**	
Protein	6710
Water	40
Chromophore	64

Other ligands	0
**B-factors (Å^2)**	
Protein	73.40
Water	53.7
Chromophore	76.1
Other ligands	0
Molprobity score	2.53
PDB ID	8C0N

Values in parenthesis are for the highest-resolution shell.

TagFT-stop gene:

ATGGTGTCTAAGGGCGAAGAGCTGATTAAGGAGAACATGCACATTAAGCTGTACATGGAGGGCACCGTGAACAACCACC
ACTTCAAGTGCACATCCGAGGGCGAAGGCAAGCCCTACGAGGGCACCCAGACCATGAGAGTCAAGGTGGTCGAGGGCGG
CCCTCTCCCCTTCGCCTTCGACATCCTGGCTACCAGCTTCTTGTACGGCAGCAGGACCTTCATCAACCACACCCAGGGCATC
CCCGACTACTTTAAGCAGTCCTTCCCTGAGGGCTTCACATGGGAGAGAGTCACCACATACGAAGACGGGGGCGTGCTGAC
CGCTACCCAGGACACCAGCCTCCAGGACGGCTGCCTCATCTACAACGTCAAGATCAGAGGGGTGGACTTCCCATCCAACG
GCCCTGTGATGCAGAAGAAAACACTCGGCTGGGAGGCCAGCACCGAGGTGCTGTACCCCGCTGACGGCGGCCTGGAAGG
CAGAGCCGTCATGGCCCTGAGGCTCGTGGGCGGGGGCCACCTGATCTGCAACCTCAAGACCACTTACAGATCCAGGAAAC
CCGCAAAGAGCCTCAAGATGCCCGGCGTCTACTATGTGGACCACAGACTGGAGAGAATCAAGGAGGCCGACAAAGAGAC
CTACGTCGAGCTGCACGAGGTGGCTGTGGCCAGATACTGCGACCTCCCTAGCAAACTGGGGCACAGATAA

mTagFT-stop gene:

ATGGTGTCTAAGGGCGAAGAGCTGATTAAGGAGATCATGCACATTAAGCTGTACATGGAGGGCACCGTGAATAACCACCACTTCA
AGTGCACATCCGAGGGCGAAGGCAAGCCCTACGAGGGCACCCAGACCATGAGAGTCAAGGTGGTCGAGGGCGGCCCTCTCCCCT
TCGCCTTCGACATCCTGGCTACCAGCTTCTTGTACGGCAGCAGGACCTTTATCAACCACACCCAGGGCATCCCCGACTTTttTAAGC
AGTCCTTCCCTGAGGGCTTCACATGGGAGAGAGTCACCACATACGAAGACGGGgGCGtGCTGACCGCTACCCAGGACACCAGCCT
CCAGGACGGCTGCCTCATCTACAACGTCAAGATCAGAGGGgTGAACTTCCCATCCAACGGCCCTGTGATGCAGAAGAAAACACTC
GGCTGGGAGGCCAGCACCGAGAAACTGAAGCCCGCTGACGGCGGCCTGGAAGGCAGAAGCGACATGGCCCTGAAGCTCGTGGGC
GGGGGCCACCTGATCTGCAACTTTAAGACCACATACAGATCCAAGAAACCCGCTAAGAACCTCAAGATGCCCGGCGTCTACTATG
TGGACCACAGACTGGAAAGAATCAAGGAGGCCGACAAAGAGACCTACGTCGAGCTGCACGAGGTGTCTGTAGCCAGATACCCGG
ACCTCCCTAGCAAACTGGGGCACAGATAA

mTsFT-stop gene:

ATGAGCGAGCTGATTAAGGAGAACATGCACATGAAGCTGTACATGGAGGGCACCGTGGACAACCATCACTTCAAGTGCAC
ATCCGAGGGCGAAGGCAAGCCCTACGAGGGCACCCAGACCATGAGAATCAAGGTGGTCGAGGGCGGCCCTCTCCCCTTCG
CCTTCGACATCCTGGCTACTAGCTTCCTCTACGGCAGCAAGACCTTCATCAACCACACCCAGGGCATCCCCGACTTCTTCAA
GCAGTCCTTCCCTGAGGGCTTCACATGGGAGAGAGTCACCACATACGAAGACGGGGGCGTGCTGACCGCTACCCAGGACA
CCAGCCTCCAGGACGGCTGCCTCATCTACAACGTCAAGATCAGAGGGGTGAACTTCACATCCAACGGCCCTGTGATGCAG
AAGAAAACACTCGGCTGGGAGGCCTTCACCGAGACGCTGTACCCCGCTGACGGCGGCCTGGAAGGCAGAAACGACATGG
CCCTGAGGCTCGTGGGCGGGAGCCATCTGATCGCAAACGCCAAGACCACATATAGATCCAAGAAACCCGCTAAGAACCTC
AAGATGCCTGGCGTCTACTATGTGGACTACAGACTGGAAAGAATCAAGGAGGCCAACAACGAGACCTACGTCGAGCAGC
ACGAGGTGGCAGTGGCCAGATACTGCGACCTCCCTAGCaaactggggcacaagctcaatgaattcATGGTGAGCAAGGGCGAGGCAGTG
ATCAAGGAGTTCATGCGGTTCAAGGTGCACATGGAGGGCTCCATGAACGGCCACGAGTTCGAGATCGAGGGCGAGGGCG
AGGGCCGCCCCTACGAGGGCACCCAGACCGCCAAGCTGAAGGTGACCAAGGGTGGCCCCCTGCCCTTCTCCTGGGACATC
CTGTCCCCTCAGTTCATGTACGGCTCCAGGGCCTTCACCAAGCACCCCGCCGACATCCCCGACTACTATAAGCAGTCCTTCC
CCGAGGGCTTCAAGTGGGAGCGCGTGATGAACTTCGAGGACGGCGGCGCCGTGACCGTGACCCAGGACACCTCCCTGGAG
GACGGCACCCTGATCTACAAGGTGAAGCTCCGCGGCACCAACTTCCCTCCTGACGGCCCCGTAATGCAGAAGAAGACAAT
GGGCTGGGAAGCGTCCACCGAGCGGTTGTACCCCGAGGACGGCGTGCTGAAGGGCGACATTAAGATGGCCCTGCGCCTGA
AGGACGGCGGCCGCTACCTGGCGGACTTCAAGACCACCTACAAGGCCAAGAAGCCCGTGCAGATGCCCGGCGCCTACAA
CGTCGACCGCAAGTTGGACATCACCTCCCACAACGAGGACTACACCGTGGTGGAACAGTACGAACGCTCCGAGGGCCGCC
ACTCCACCGGCGGCATGGACGAGCTGTATAAGTAA

mNeptusFT2-stop gene:

ATGAGCGAGCTGATTAAGGAGAACATGCACATGAAGCTGTACATGGAGGGTACGGTGAACAACCACCACTTCAAGTGCAC
ATCCGAGGGCGAAGGCAAGCCCTACGAGGGCACCCAGACCAGCAGAATCAAGGTGGTCGAGGGCGGCCCTCTCCCCTTC
GCCTTCGACATCCTGGCTACCTGCTTCATGTACGGCAGCAAGACCTTCATCAACCACACCCAGGGCATCCCCGACTTCTTTA
AGCAGTCCTTCCCTGAGGGCTTCACATGGGAGAGAGTCACCACATACGAAGACGGGGGCGTGCTGACCGCTACCCAGGAC
ACCAGCCTCCAGGACGGCTGCCTCATCTACAACGTCAAGATCAGAGGGGTGAACTTCCCATCCAACGGCCCTGTGATGCA
GAAGAAAACACTCGGCTGGGAGGCCTCCACCGAGACGCTGTACCCCGCTGACGGCGGCCTGGAAGGCAGATGCAACATG
GCCCTGAAGCTCGTGGGCGGGGGCCACCTGATCTGCAACTTGAAGACCACATACAGATCCAAGAAACCCGCTAAGAACCT
CAAGATGCCCGGCGTCTACTTCGTGGACCGCAGACTGGAAAGAATCAAGGAGGCCGACAAAGAGACCTATGTCGAGCAG
CACGAGGTGGCTGTGGCCAGATACTGCGACCTCCCTAGCAAACTCGGCCATAAGCTCAATGAATTCATGGTGAGCAAGGG
CGAGGAGCTGTTCACCGGGGTGGTGCCCATCCTGGTCGAGCTGGACGGCGACGTAAACGGCCACAAGTTCAGCGTGCGCG

GCGAGGGCGAGGGCGATGCCACCAACGGCAAGCTGACCCTGAAGTTCATCTGCACCACCGGCAAGCTGCCCGTGCCCTGG
CCCACCCTCGTGACCACCCTGACCTACGGCGTGCAGTGCTTCAGCCGCTACCCCGACCACATGAAGCGCCACGACTTCTTC
AAGTCCGCCATGCCCGAAGGCTACGTCCAGGAGCGCACCATCAGCTTCAAGGACGACGGCACCTACAAGACCCGCGCCG
AGGTGAAGTTCGAGGGCGACACCCTGGTGAACCGCATCGAGCTGAAGGGCATCGACTTCAAGGAGGACGGCAACATCCT
GGGGCACAAGCTGGAGTACAACTTCAACAGCCACAACGTCTATATCACCGCCGACAAGCAGAAGAACGGCATCAAGGCC
AACTTCAAGATCCGCCACAACGTGGAGGACGGCAGCGTGCAGCTCGCCGACCACTACCAGCAGAACACCCCCATCGGCG
ACGGCCCCGTGCTGCTGCCCGACAACCACTACCTGAGCACCCAGTCCGTGCTGAGCAAAGACCCCAACGAGAAGCGCGAT
CACATGGTCCTGCTGGAGTTCGTGACCGCCGCCGGGATCACTCACGGCATGGACGAGCTGTACAAGTAA

TagFT-hCdt1(1-100) gene:

ATGGCCGAAGGGAGCGTCGCAAGACAGCCTGACCTGCTGACCTGCGATGACGAACCTATTCACATTCCTGGGGCAATCCA
GCCTCACGGGCTGCTGCTGGCTCTGGCCGCTGACATGACTATCGTGGCAGGCTCTGATAACCTGCCAGAGCTGACCGGACT
GGCTATCGGGGCACTGATTGGACGATCCGCAGCAGACGTGTTCGATTCTGAGACTCACAATCGACTGACCATCGCCCTGGC
TGAACCTGGAGCTGCAGTGGGAGCACCAATTACCGTCGGCTTCACAATGAGAAAGGACGCTGGCTTTATCGGAAGTTGGC
ACCGGCATGATCAGCTGATTTTTCTGGAGCTGGAACCACCTCAGCGAGACGTGGCAGAGCCACAGGCTTTCTTTAGGAGAA
CAAACAGCGCCATCCGGCGCCTGCAGGCAGCTGAGACTCTGGAATCCGCTTGCGCAGCCGCTGCACAGGAAGTGAGGAA
GATCACCGGATTCGATCGCGTCATGATCTACAGGTTCGCCTCAGACTTTAGCGGGGAAGTGATCGCAGAAGATAGATGTGC
CGAGGTCGAAAGTAAACTGGGCCTGCATTACCCAGCCTCAACCGTGCCTGCACAGGCACGAAGGCTGTATACAATCAACC
CAGTCAGGATCATTCCCGACATTAATTACAGACCAGTGCCCGTCACACCTGACCTGAACCCAGTGACTGGACGGCCCATCG
ATCTGTCTTTCGCCATTCTGCGCAGCGTGTCCCCTGTCCACCTGGAGTTTATGCGCAATATCGGGATGCATGGCACCATGTCT
ATCAGTATTCTGAGAGGCGAACGGCTGTGGGGACTGATTGTGTGCCACCATCGAACACCTTACTATGTCGACCTGGATGGC
AGGCAGGCTTGCGAGCTGGTGGCACAGGTGCTGGCTTGGCAGATTGGAGTCATGGAGGAGCGACCGGTAGATCTTATGGA
GCAGCGCCGCGTCACCGACTTCTTCGCGCGCCGCCGCCCCGGGCCCCCCCGCATCGCGCCGCCCAAGCTGGCCTGCCGCAC
CCCCAGCCCCGCCAGGCCCGCACTCCGCGCCCCGGCCTCCGCTACCAGTGGCAGCCGCAAGCGCGCCCGCCCGCCCGCCG
CCCCCGGACGCGACCAGGCCAGGCCACCGGCCCGCAGGAGACTGCGGCTGTCGGTGGACGAGGTTTCCAGCCCCAGTACC
CCCGAGGCCCCAGACATCCCAGCCTGCCCTTCTCCGGGCCAGAAGATAAAGAAATAA

mNeptusFT2-hGeminin(1-110) gene:

ATGAGCGAGCTGATTAAGGAGAACATGCACATGAAGCTGTACATGGAGGGTACGGTGAACAACCACCACTTCAAGTGCAC
ATCCGAGGGCGAAGGCAAGCCCTACGAGGGCACCCAGACCAGCAGAATCAAGGTGGTCGAGGGCGGCCCTCTCCCCTTC
GCCTTCGACATCCTGGCTACCTGCTTCATGTACGGCAGCAAGACCTTCATCAACCACACCCAGGGCATCCCCGACTTCTTTA
AGCAGTCCTTCCCTGAGGGCTTCACATGGGAGAGAGTCACCACATACGAAGACGGGGGCGTGCTGACCGCTACCCAGGAC
ACCAGCCTCCAGGACGGCTGCCTCATCTACAACGTCAAGATCAGAGGGGTGAACTTCCCATCCAACGGCCCTGTGATGCA
GAAGAAAACACTCGGCTGGGAGGCCTCCACCGAGACGCTGTACCCCGCTGACGGCGGCCTGGAAGGCAGATGCAACATG
GCCCTGAAGCTCGTGGGCGGGGGCCACCTGATCTGCAACTTGAAGACCACATACAGATCCAAGAAACCCGCTAAGAACCT
CAAGATGCCCGGCGTCTACTTCGTGGACCGCAGACTGGAAAGAATCAAGGAGGCCGACAAAGAGACCTATGTCGAGCAG
CACGAGGTGGCTGTGGCCAGATACTGCGACCTCCCTAGCAAACTCGGCCATAAGCTCAATGAATTCATGGTGAGCAAGGG
CGAGGAGCTGTTCACCGGGGTGGTGCCCATCCTGGTCGAGCTGGACGGCGACGTAAACGGCCACAAGTTCAGCGTGCGCG
GCGAGGGCGAGGGCGATGCCACCAACGGCAAGCTGACCCTGAAGTTCATCTGCACCACCGGCAAGCTGCCCGTGCCCTGG
CCCACCCTCGTGACCACCCTGACCTACGGCGTGCAGTGCTTCAGCCGCTACCCCGACCACATGAAGCGCCACGACTTCTTC
AAGTCCGCCATGCCCGAAGGCTACGTCCAGGAGCGCACCATCAGCTTCAAGGACGACGGCACCTACAAGACCCGCGCCG
AGGTGAAGTTCGAGGGCGACACCCTGGTGAACCGCATCGAGCTGAAGGGCATCGACTTCAAGGAGGACGGCAACATCCT
GGGGCACAAGCTGGAGTACAACTTCAACAGCCACAACGTCTATATCACCGCCGACAAGCAGAAGAACGGCATCAAGGCC
AACTTCAAGATCCGCCACAACGTGGAGGACGGCAGCGTGCAGCTCGCCGACCACTACCAGCAGAACACCCCCATCGGCG
ACGGCCCCGTGCTGCTGCCCGACAACCACTACCTGAGCACCCAGTCCGTGCTGAGCAAAGACCCCAACGAGAAGCGCGAT
CACATGGTCCTGCTGGAGTTCGTGACCGCCGCCGGGATCACTCACGGCATGGACGAGCTGTACAAGCGACCGGTTATGAAT
CCCAGTATGAAGCAGAAACAAGAAGAAATCAAAGAGAATATAAAGAATAGTTCTGTCCCAAGAAGAACTCTGAAGATGA
TTCAGCCTTCTGCATCTGGATCTCTTGTTGGAAGAGAAAATGAGCTGTCCGCAGGCTTGTCCAAAAGGAAACATCGGAATG
ACCACTTAACATCTACAACTTCCAGCCCTGGGGTTATTGTCCCAGAATCTAGTGAAAATAAAAATCTTGGAGGAGTCACCC
AGGAGTCATTTGATCTTATGATTAAAGAAAATCCATCCTCTCAGTATTGGAAGGAAGTGGCAGAAAAACGGAGAAAGGCG
CTGTAA

SARE-ArcMin-TagFT-3xNLS gene:

GTGGCAGGCTCAGCGCACAGAGCCTTCCTGCGTGGGGAAGCTCCTTGCTGCGTCATGGCTCAGCTATTCTCAGCCTCTCTCC
TTTTATGGTGCCGGAAGCAGGCAGGCTGCTGCTGGGCTGGCTCGGTGGGAGGCGCGCAGCAGAGCACATTAGTCACTCGG
GGCTGTGAAGGGGCGGGTCCTTGAGGGCACCCACGGGAGGGGAGCGAGTAGGCGCGGAAGGCGGGGCCTGCGGCAGGA
GAGGGCGCGGGCGGGCTCTGGCGCGGAGCCTGGGCGCCGCCAATGGGAGCCAGGGCTCCACGAGCTGCCGCCCACGGGC
CCCGCGCAGCATAAATAGCCGCTGGTGGCGGTTTCGGTGCAGAGCTCAAGCGAGTTCTCCCGCAGCCGCAGTCTCTGGGCC
TCTCTAGCTTCAGCGGCGACGAGCCTGCCACACTCGCTAAGCTCCTCCGGCACCGCACACCTGCCACTGCCGCTGCAGCCG
CCGGCTCTGCTCCCTTCCGGCTTCTGCCTCAGAGGAGTTCTTAGCCTGTTCGGAGCCGCAGCACCGACGACCAGATGTCTAG
AGATATCATGGTGTCTAAGGGCGAAGAGCTGATTAAGGAGAACATGCACATTAAGCTGTACATGGAGGGCACCGTGAACA
ACCACCACTTCAAGTGCACATCCGAGGGCGAAGGCAAGCCCTACGAGGGCACCCAGACCATGAGAGTCAAGGTGGTCGA
GGGCGGCCCTCTCCCCTTCGCCTTCGACATCCTGGCTACCAGCTTCTTGTACGGCAGCAGGACCTTCATCAACCACACCCAG
GGCATCCCCGACTACTTTAAGCAGTCCTTCCCTGAGGGCTTCACATGGGAGAGAGTCACCACATACGAAGACGGGGGCGT
GCTGACCGCTACCCAGGACACCAGCCTCCAGGACGGCTGCCTCATCTACAACGTCAAGATCAGAGGGGTGGACTTCCCAT
CCAACGGCCCTGTGATGCAGAAGAAAACACTCGGCTGGGAGGCCAGCACCGAGGTGCTGTACCCCGCTGACGGCGGCCTG
GAAGGCAGAGCCGTCATGGCCCTGAGGCTCGTGGGCGGGGGCCACCTGATCTGCAACCTCAAGACCACTTACAGATCCAG
GAAACCCGCAAAGAGCCTCAAGATGCCCGGCGTCTACTATGTGGACCACAGACTGGAGAGAATCAAGGAGGCCGACAAA
GAGACCTACGTCGAGCTGCACGAGGTGGCTGTGGCCAGATACTGCGACCTCCCTAGCAAACTGGGGCACAGATCCGGAC
TCAGATCTCGAGCTGATCCAAAAAAGAAGAGAAAGGTAGATCCAAAAAAGAAGAGAAAGGTA
GATCCAAAAAAGAAGAGAAAGGTAGGATCCACCGGATCTAGATAA

Figure S1. Nucleotide sequences of the TagFT, mTagFT, mTsFT and mNeptusFT2 proteins, their fusions with hCdt1(1-100) and hGeminin(1-110) proteins and Arc1 (SARE-ArcMin) promoter. Arc1 promoter is assembled from SARE and ArcMin which are 125 bp SARE regulatory element from -7kb region of arc promoter and minimal arc promoter, respectively (both cloned from genomic DNA isolated from C57/Bl6 mouse). 3xNLS is three repeats of the nuclear localization signal.

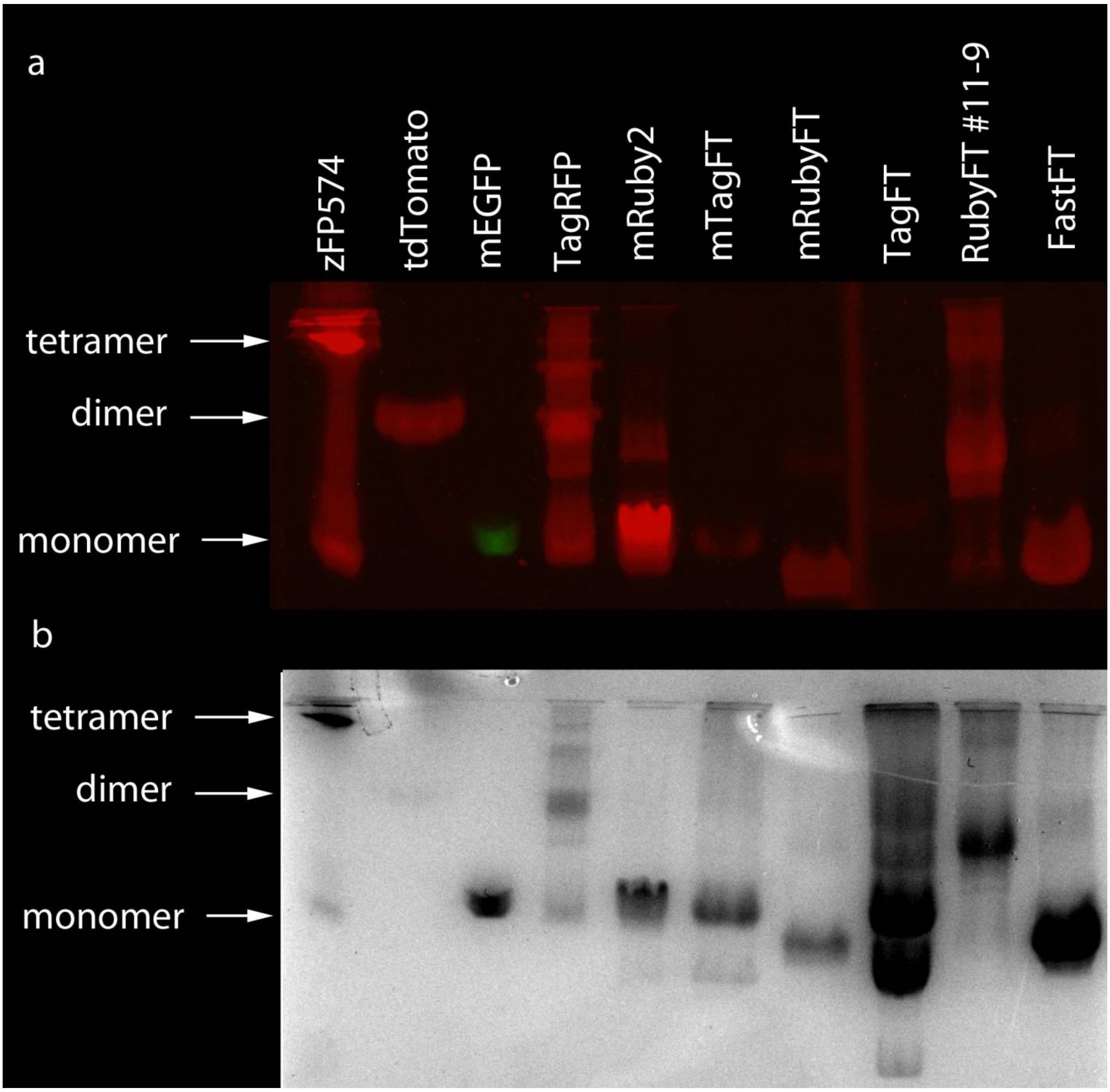

Figure S2. Semi-native polyacrylamide gel with TagFT and mTagFT timers and control zFP754, tdTomato, mEGFP, TagRFP, mRuby2, mRubyFT, RubyFT#11-9 and FastFT proteins. 100-235 μg of freshly purified fluorescent proteins were loaded in 20 μl aliquots onto a semi-native 12.5% polyacrylamide gel containing 0.5% sodium dodecyl sulfate (SDS). The gel was run at 100 Volts, 25 °C. mEGFP, tdTomato and zFP574 were loaded as monomer, dimer and tetramer protein standards, respectively. (a) In the fluorescent channels, the gel was photographed using a Leica M205FA fluorescence stereomicroscope. (b) After fixation and staining with 1% Panseau S, the gel was photographed in visible light using the G: Box Chemi-XT4 GENESys system.

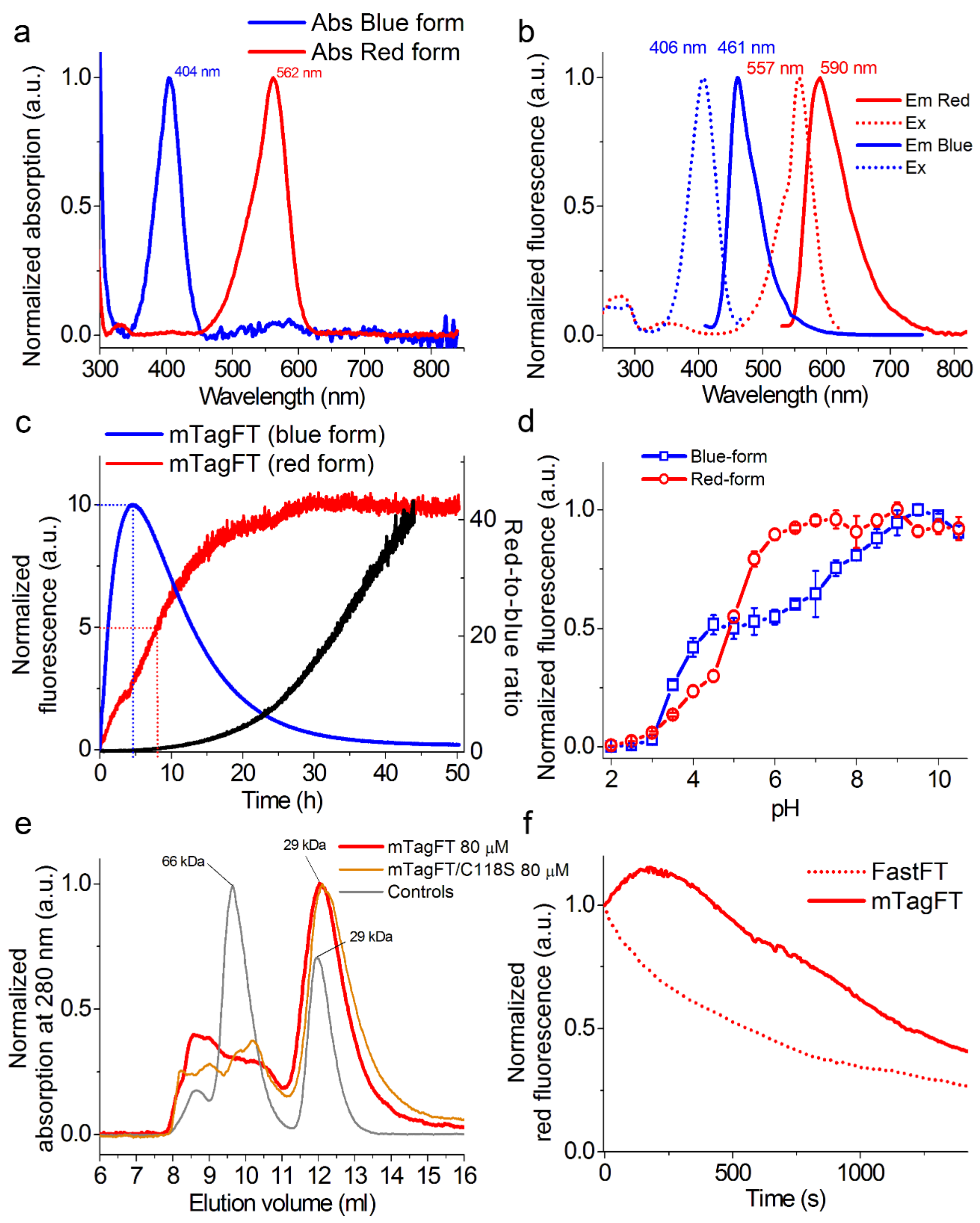

Figure S3. In vitro properties of the purified mTagFT protein. (**a**) Absorption spectra for blue and red forms of mTagFT protein in PBS buffer at pH 7.40. (**b**) Excitation and emission spectra for blue and red forms of mTagFT in PBS buffer at pH 7.40. (**c**) Maturation of blue and red forms for mTagFT in PBS buffer at pH 7.40, 37 °C. Red-to-blue ratio was calculated according to the red and blue fluorescence time dependences normalized to 100. (**d**) Fluorescence intensity for blue and red forms of mTagFT as a function of pH. Three replicates were averaged for analysis. Error bars represent the standard deviation. (**e**) Fast protein liquid chromatography of mTagFT protein and its mTagFT/C118S mutant. Proteins were eluted in 20 mM Tris-HCl (pH 7.80) and 200 mM NaCl buffer. The molecular weight of mTagFT and its mutant were calculated from a linear regression of the dependence of logarithm of control molecular weights vs. elution volume. (**f**) Photostability of red forms for mTagFT and control FastFT timers under continuous wide-field imaging using a mercury lamp (9 mW/cm^2 550/25BP light power before objective lens).

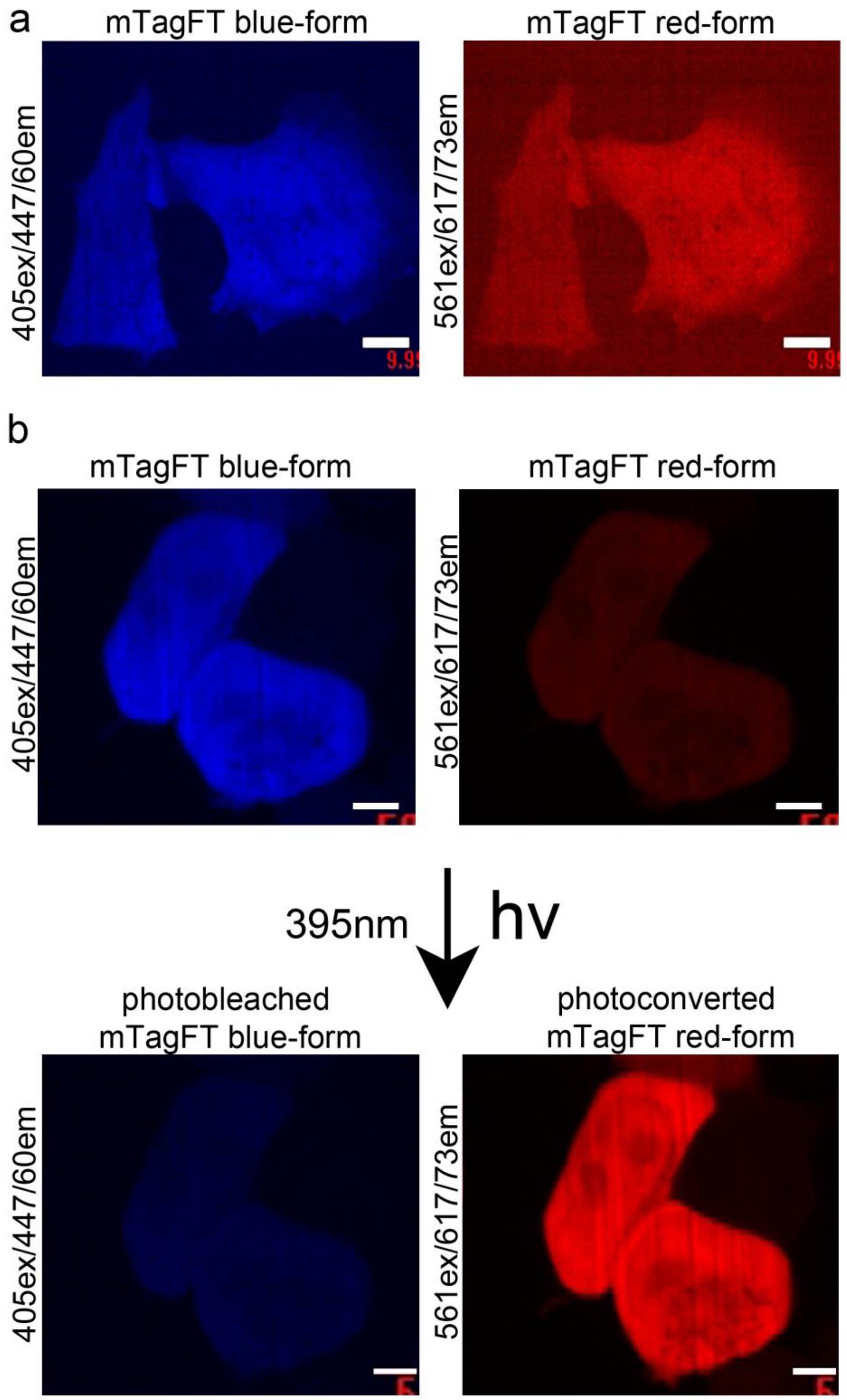

Figure S4. Localization and blue-to-red photoconversion for the mTagFT timer in live mammalian cells. (**a**) Confocal images of live HeLa cells expressing mTagFT-P2A-EGFP fusion. P2A is a self-cleavable peptide. Blue (405ex and 447/60em), and red (561ex and 617/73em) fluorescence channels are shown for expression lasted for 72h. (**b**) Confocal images of live HEK293T cells expressing mTagFT-P2A-EGFP fusion. Blue (405ex and 447/60em) and red (561ex and 617/73em) fluorescence channels before and after continuous irradiation with 395/25 nm light for 1 min are shown. Protein expression lasted 24 h. For red and blue images, the contrast settings were the same before and after photoconversion. Images were acquired 72 h (**a**) or 24 h (**b**) after transfection. (**a,b**) Scale bars: 10 μm (a) and 50 μm (b).

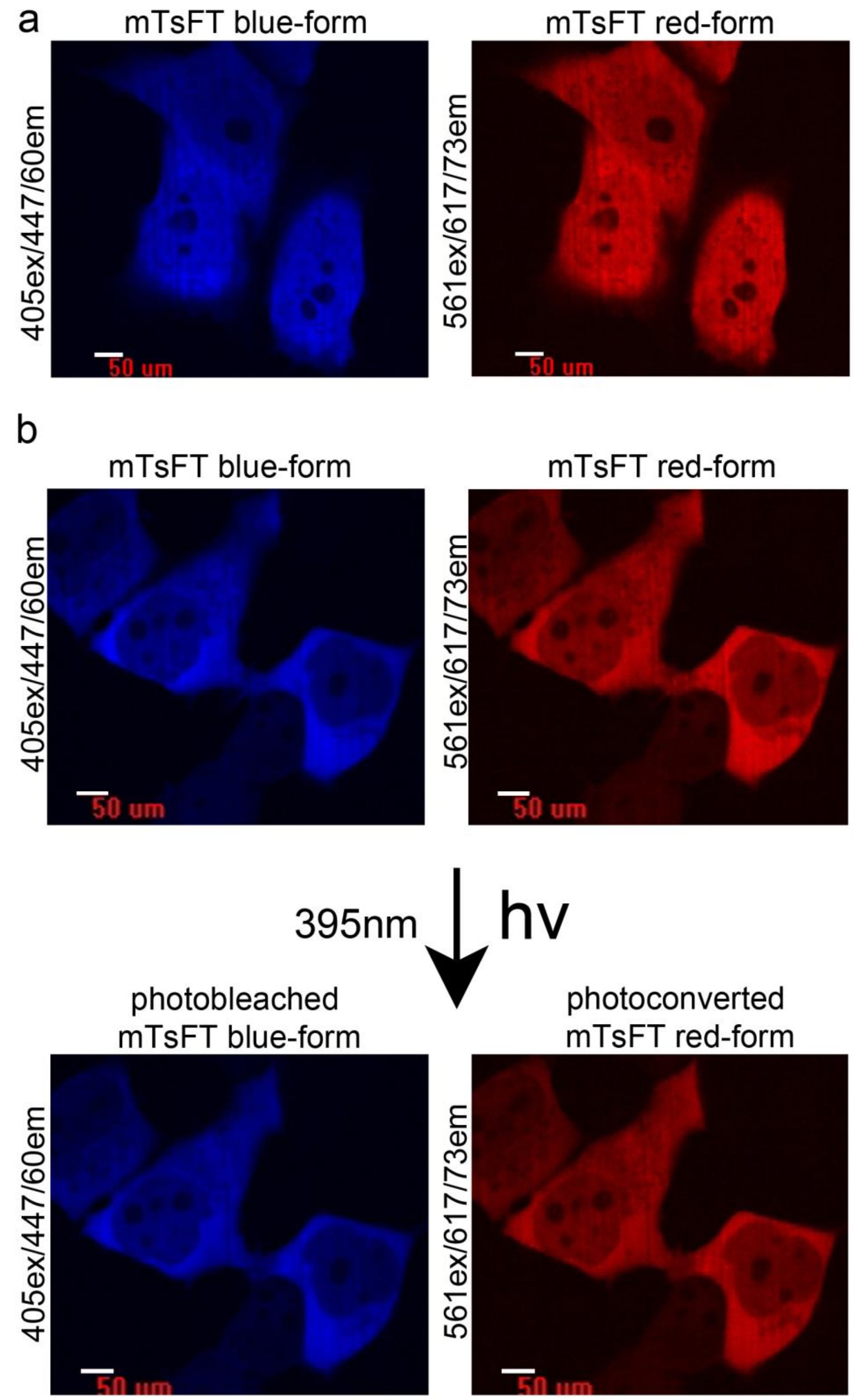

Figure S5. Localization and blue-to-red photoconversion for the mTsFT timer in live mammalian cells. (**a**) Confocal images of live HeLa cells expressing mTsFT-P2A-EGFP fusion. P2A is a self-cleavable peptide. Blue (405ex and 447/60em), and red (561ex and 617/73em) fluorescence channels are shown for expression lasted for 72h. (**b**) Confocal images of live HEK293T cells expressing mTsFT-P2A-EGFP fusion. Blue (405ex and 447/60em) and red (561ex and 617/73em) fluorescence channels before and after continuous irradiation with 395/25 nm light for 1 min are shown. Protein expression lasted 24 h. For red and blue images, the contrast settings were the same before and after photoconversion. Images were acquired 72 h (**a**) or 24 h (**b**) after transfection. (**a**,**b**) Scale bars: 50 μm.

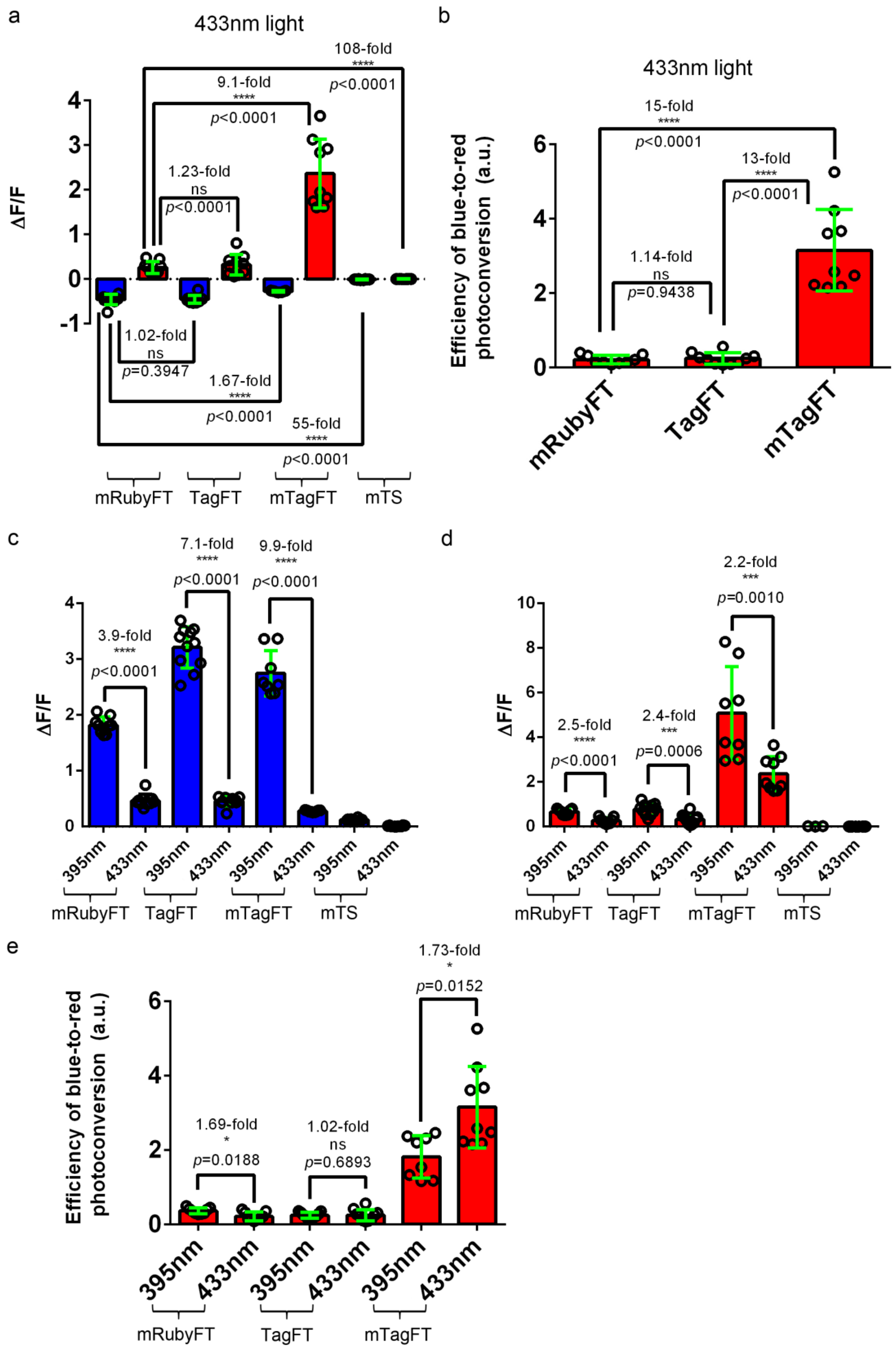

Figure S6. Comparison of the photoconversion of TagFT, mTagFT, mTS and control mRubyFT timers with blue 395/25 nm (0.338 mW/cm² before objective lens) and 433/25 nm light (0.920 mW/cm² before objective lens) in live HEK293T cells. The values of ΔF/F and efficiency of photoconversion were normalized to the light power. **(a)** The mean ΔF/F values for the photobleached blue form (blue bars) and photoconverted red form (red bars) of TagFT, mTagFT, mTsFT and control mRubyFT timers expressed in

live HEK293T cells 24 h after transfection. The pulse of 433/25 mn light lasted for 1 min. (**b**) The efficiency of the blue-to-red photoconversion with 433/25 nm light for 1 min was calculated as $\Delta F/F_{red}/\Delta F/F_{blue}$. (**c,d**) The values of $\Delta F/F$ for photoconversion of blue forms (**c**) and red forms (**d**) using 395 or 433 nm light. (**e**) The efficiency of photoconversion with 395 or 433 nm light.

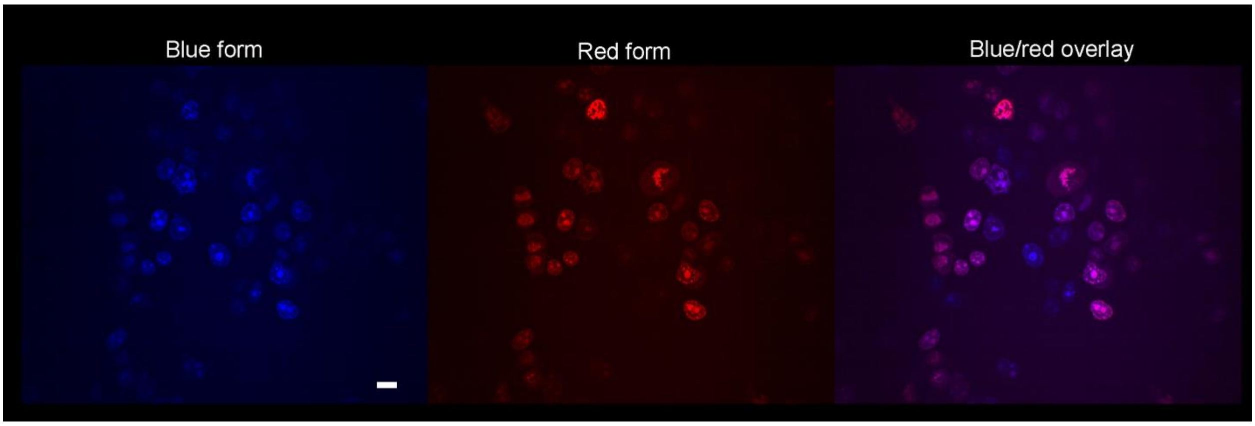

Figure S7. Expression of the blue-to-red TagFT fluorescent timer under the control of the minimal Arc1 promoter in HEK293T cells. Confocal images of the cells 24 hours after transfection with pAAV-SARE-ArcMin-TagFT-3xNLS plasmid in blue (405ex/447/60em), red (560ex/617/73em), and superimposed blue-red channels. Scale bar: 15 μm.

```
mNeptus      1:MSELIKENMHMKLYMEGTVNNHHFKCTSEGEGKPYEGTQTGRIKVVEGGPLPFAFDILAT 60
mNeptusFT1   1:MSELIKENMHMKLYMEGTVNNHHFKCTSEGEGKPYEGTQTGRIKVVEGGPLPFAFDILAT 60
mNeptusFT2   1:MSELIKENMHMKLYMEGTVNNHHFKCTSEGEGKPYEGTQTGRIKVVEGGPLPFAFDILAT 60
                 ***
mNeptus     61:CFMYGSKTFINHTQGIPDFFKQSFPEGFTWERVTTYEDGGVLTATQDTSLQDGCLIYNVK 120
mNeptusFT1  61:CFMYGSKTFINHTQGIPDFFKQSFPEGFTWERVTTYEDGGVLTATQDTSLQDGCLIYNVK 120
mNeptusFT2  61:CFMYGSKTFINHTQGIPDFFKQSFPEGFTWERVTTYEDGGVLTATQDTSLQDGCLIYNVK 120

mNeptus    121:IRGVNFPSNGPVMQKKTLGWEASTETLYPADGGLEGRCDMALKLVGGGHLICNLKTTYRS 180
mNeptusFT1 121:IRGVNFPSNGPVMQKKTLGWEASTETLYPADGGLEGRCDMALKLVGGGHLICNLKTTYRS 180
mNeptusFT2 121:IRGVNFPSNGPVMQKKTLGWEASTETLYPADGGLEGRCNMALKLVGGGHLICNLKTTYRS 180

mNeptus    181:KKPAKNLKMPGVYFVDRRLERIKEADKETYVEQHEVAVARYCDLPSKLGHKLNEFMVSKG 240
mNeptusFT1 181:KKPAKNLKMPGVYFVDRRLERIKEADKETYVEQHEVAVARYCDLPSKLGHKLNEFMVSKG 240
mNeptusFT2 181:KKPAKNLKMPGVYFVDRRLERIKEADKETYVEQHEVAVARYCDLPSKLGHKLNEFMVSKG 240

mNeptus    241:EELFTGVVPILVELDGDVNGHKFSVRGEGEGDATNGKLTLKFICTTGKLPVPWPTLVTTL 300
mNeptusFT1 241:EELFTGVVPILVELDGDVNGHKFSVRGEGEGDATNGKLTLKFICTTGKLPVPWPTLVTTL 300
mNeptusFT2 241:EELFTGVVPILVELDGDVNGHKFSVRGEGEGDATNGKLTLKFICTTGKLPVPWPTLVTTL 300
               ***
mNeptus    301:TYGVQCFSRYPDHMKRHDFFKSAMPEGYVQERTISFKDDGTYKTRAEVKFEGDTLVNRIE 360
mNeptusFT1 301:TYGVQCFSRYPDHMKRHDFFKSAMPEGYVQERTISFKDDGTYKTRAEVKFEGDTLVNRIE 360
mNeptusFT2 301:TYGVQCFSRYPDHMKRHDFFKSAMPEGYVQERTISFKDDGTYKTRAEVKFEGDTLVNRIE 360

mNeptus    361:LKGIDFKEDGNILGHKLEYNFNSHNVYITADKQKNGIKANFKIRHNVEDGSVQLADHYQQ 420
mNeptusFT1 361:LKGIDFKEDGNILGHKLEYNFNSHNVYITADKQKNGIKANFKIRHNVEDGSVQLADHYQQ 420
mNeptusFT2 361:LKGIDFKEDGNILGHKLEYNFNSHNVYITADKQKNGIKANFKIRHNVEDGSVQLADHYQQ 420

mNeptus    421:NTPIGDGPVLLPDNHYLSTQSVLSKDPNEKRDHMVLLEFVTAAGITHGMDELYK       474
mNeptusFT1 421:NTPIGDGPVLLPDNHYLSTQSVLSKDPNEKRDHMVLLEFVTAAGITHGMDELYK       474
mNeptusFT2 421:NTPIGDGPVLLPDNHYLSTQSVLSKDPNEKRDHMVLLEFVTAAGITHGMDELYK       474
```

Figure S8. Amino acid sequence alignment for green-to-far-red tandem timer based on mNeptune-sfGFP fusion (mNeptus). mNeptus is the original timer. The mNeptune and sfGFP proteins are shown in far-red and green, respectively. Residues inside the β-barrel of the mNeptune protein are shown in grey. Asterisks indicate residues that form chromophores. External mutation relative to the original mNeptus template is highlighted in blue.

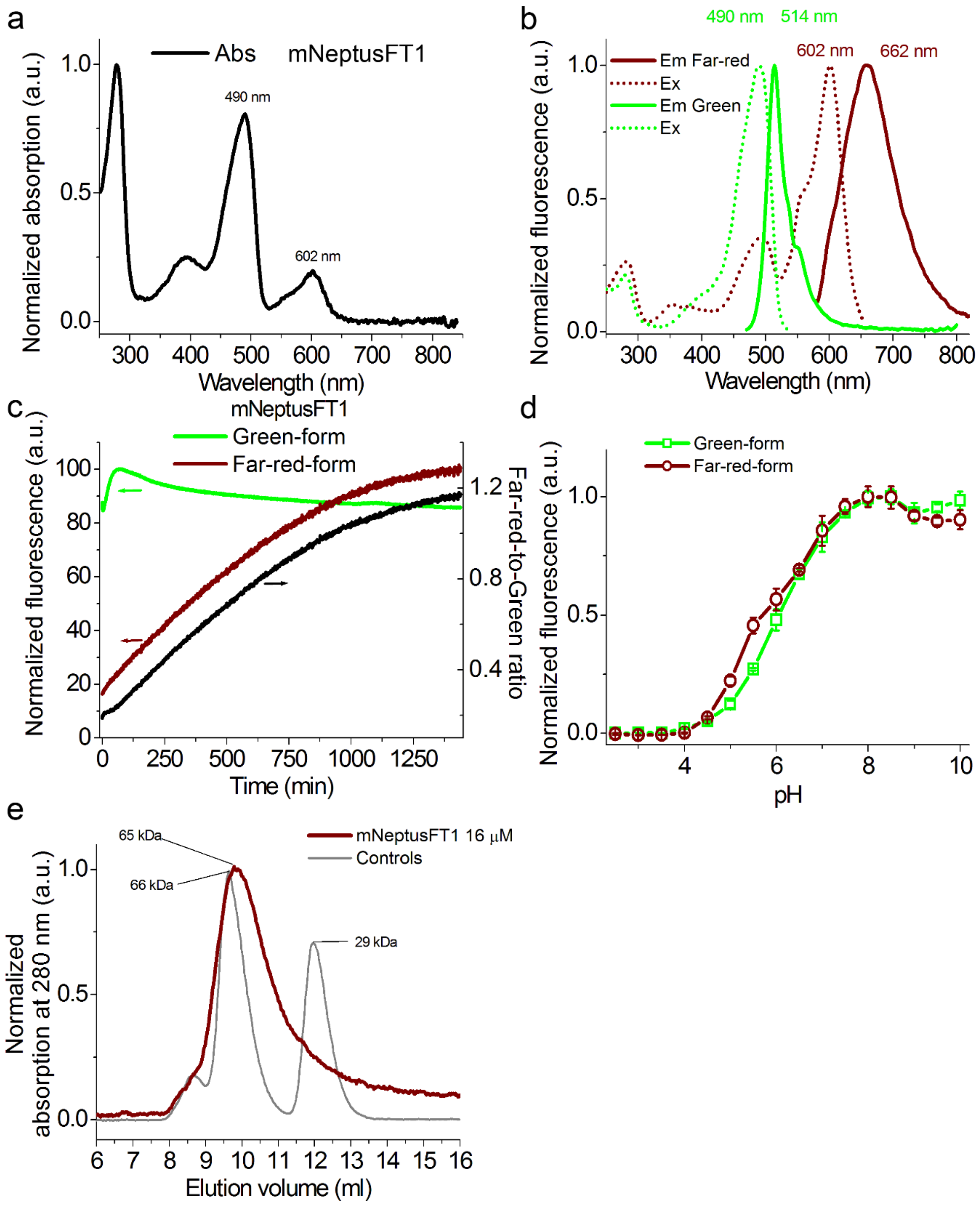

Figure S9. In vitro properties of the purified mNeptusFT1 protein. (**a**) Absorption spectra for green and far-red forms of mNeptusFT1 protein in PBS buffer at pH 7.40. (**b**) Excitation and emission spectra for green and far-red forms of mNeptusFT1 in PBS buffer at pH 7.40. (**c**) Maturation of green and far-red forms for mNeptusFT1 in PBS buffer at pH 7.40, 37 °C. Far-red-to-green ratio was calculated according to the far-red and green fluorescence time dependences normalized to 100. (**d**) Fluorescence intensity for green and far-red forms of mNeptusFT1 as a function of pH. Three replicates were averaged for analysis. Error bars represent the standard deviation. (**e**) Fast protein liquid chromatography of mNeptusFT1 protein. Proteins were eluted in 20 mM Tris-HCl (pH 7.80) and 200 mM NaCl buffer. The molecular weight of mNeptusFT1 (57 kDa theoretical) was calculated from a linear regression of the dependence of logarithm of control molecular weights vs. elution volume.

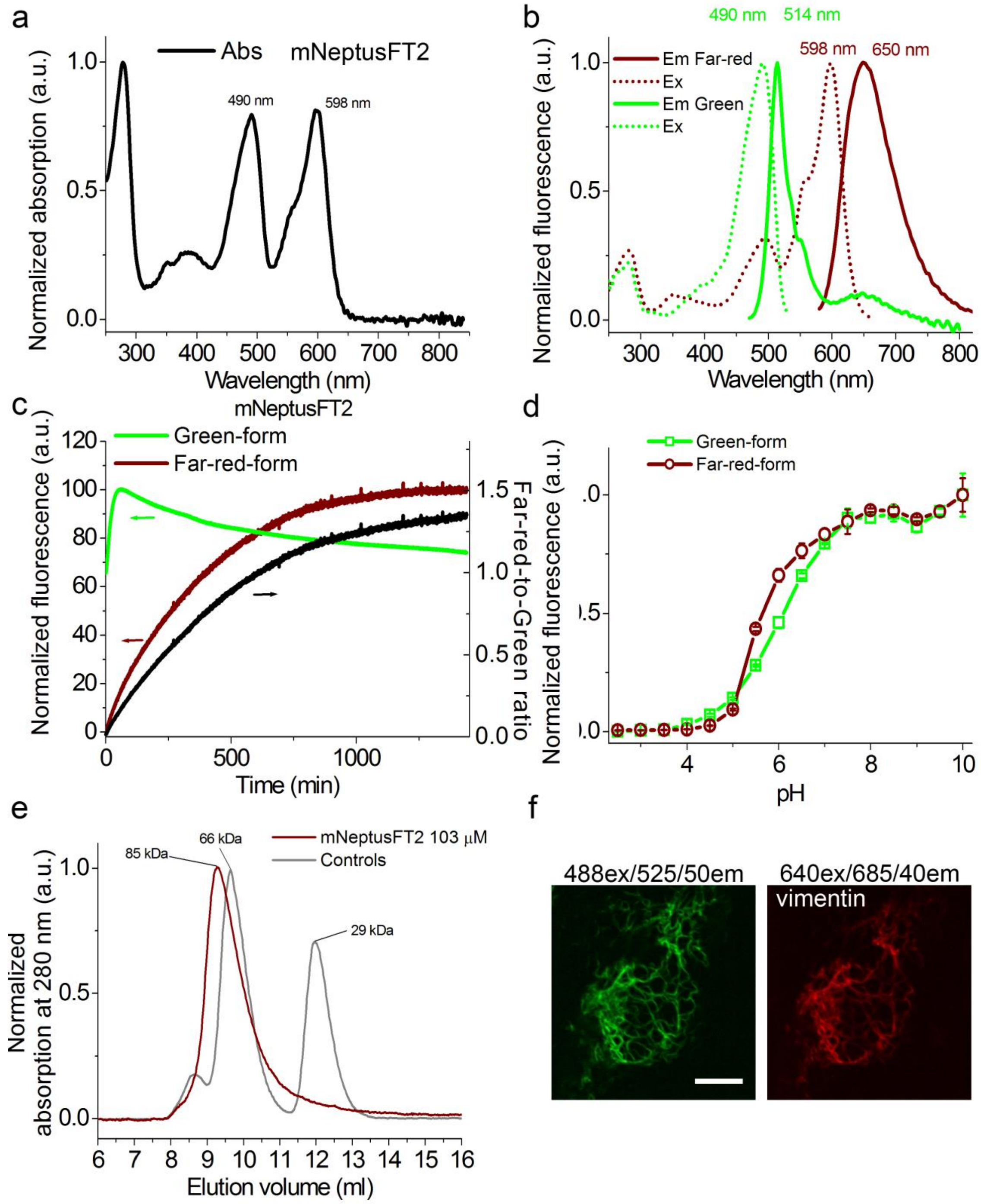

Figure S10. Properties of the purified mNeptusFT2 protein in vitro and in HeLa cells. (**a**) Absorption spectra for green and far-red forms of mNeptusFT2 protein in PBS buffer at pH 7.40. (**b**) Excitation and emission spectra for green and far-red forms of mNeptusFT2 in PBS buffer at pH 7.40. (**c**) Maturation of green and far-red forms for mNeptusFT2 in PBS buffer at pH 7.40, 37 °C. Far-red-to-green ratio was calculated according to the far-red and green fluorescence time dependences normalized to 100. (**d**) Fluorescence intensity for green and far-red forms of mNeptusFT2 as a function of pH. Three replicates were averaged for analysis. Error bars represent the standard deviation. (**e**) Fast protein liquid chromatography of mNeptusFT2 protein. Proteins were eluted in 20 mM Tris-HCl (pH 7.80) and 200 mM NaCl buffer. The molecular weight of mNeptusFT2 (57 kDa theoretical) was calculated from a linear regression of the dependence of logarithm of control molecular weights vs. elution volume. (**f**) Confocal images of HeLa Kyoto cells in green (488ex and 525/50em) and far-red (640ex and 685/40em) channels 48 h after transfection with pVimentin-mNeptusFT2 plasmid. Scale bar: 10 μm.

```
mTS         1:MSELIKENMHMKLYMEGTVDNHHFKCTSEGEGKPYEGTQTMRIKVVEGGPLPFAFDILAT 60
mTsFT       1:MSELIKENMHMKLYMEGTVDNHHFKCTSEGEGKPYEGTQTMRIKVVEGGPLPFAFDILAT 60
               ***
mTS        61:SFLYGSKTFINHTQGIPDFFKQSFPEGFTWERVTTYEDGGVLTATQDTSLQDGCLIYNVK 120
mTsFT      61:SFLYGSKTFINHTQGIPDFFKQSFPEGFTWERVTTYEDGGVLTATQDTSLQDGCLIYNVK 120

mTS       121:IRGVNFTSNGPVMQKKTLGWEAFTETLYPADGGLEGRNDMALKLVGGSHLIANAKTTYRS 180
mTsFT     121:IRGVNFTSNGPVMQKKTLGWEAFTETLYPADGGLEGRNDMALRLVGGSHLIANAKTTYRS 180

mTS       181:KKPAKNLKMPGVYYVDYRLERIKEANNETYVEQHEVAVARYCDLPSKLGHKLNEFMVSKG 240
mTsFT     181:KKPAKNLKMPGVYYVDYRLERIKEANNETYVEQHEVAVARYCDLPSKLGHKLNEFMVSKG 240

mTS       241:EAVIKEFMRFKVHMEGSMNGHEFEIEGEGEGRPYEGTQTAKLKVTKGGPLPFSWDILSPQ 300
mTsFT     241:EAVIKEFMRFKVHMEGSMNGHEFEIEGEGEGRPYEGTQTAKLKVTKGGPLPFSWDILSPQ 300
              ***
mTS       301:FMYGSRAFTKHPADIPDYYKQSFPEGFKWERVMNFEDGGAVTVTQDTSLEDGTLIYKVKL 360
mTsFT     301:FMYGSRAFTKHPADIPDYYKQSFPEGFKWERVMNFEDGGAVTVTQDTSLEDGTLIYKVKL 360

mTS       361:RGTNFPPDGPVMQKKTMGWEASTERLYPEDGVLKGDIKMALRLKDGGRYLADFKTTYKAK 420
mTsFT     361:RGTNFPPDGPVMQKKTMGWEASTERLYPEDGVLKGDIKMALRLKDGGRYLADFKTTYKAK 420

mTS       421:KPVQMPGAYNVDRKLDITSHNEDYTVVEQYERSEGRHSTGGMDELYK              467
mTsFT     421:KPVQMPGAYNVDRKLDITSHNEDYTVVEQYERSEGRHSTGGMDELYK              467
```

Figure S11. Alignment of amino acid sequences for a blue-to-red tandem timer based on the mTagBFP2-mScarlet fusion protein. mTS is the original timer. The mTagBFP2 and mScarlet parts of the timer are shown in blue and red color, respectively. Residues inside the β-barrel of the mTagBFP2 protein are highlighted in gray. Asterisks indicate residues, which form chromophores. Mutation relative to the original template in mTagBFP2 part is highlighted in blue.

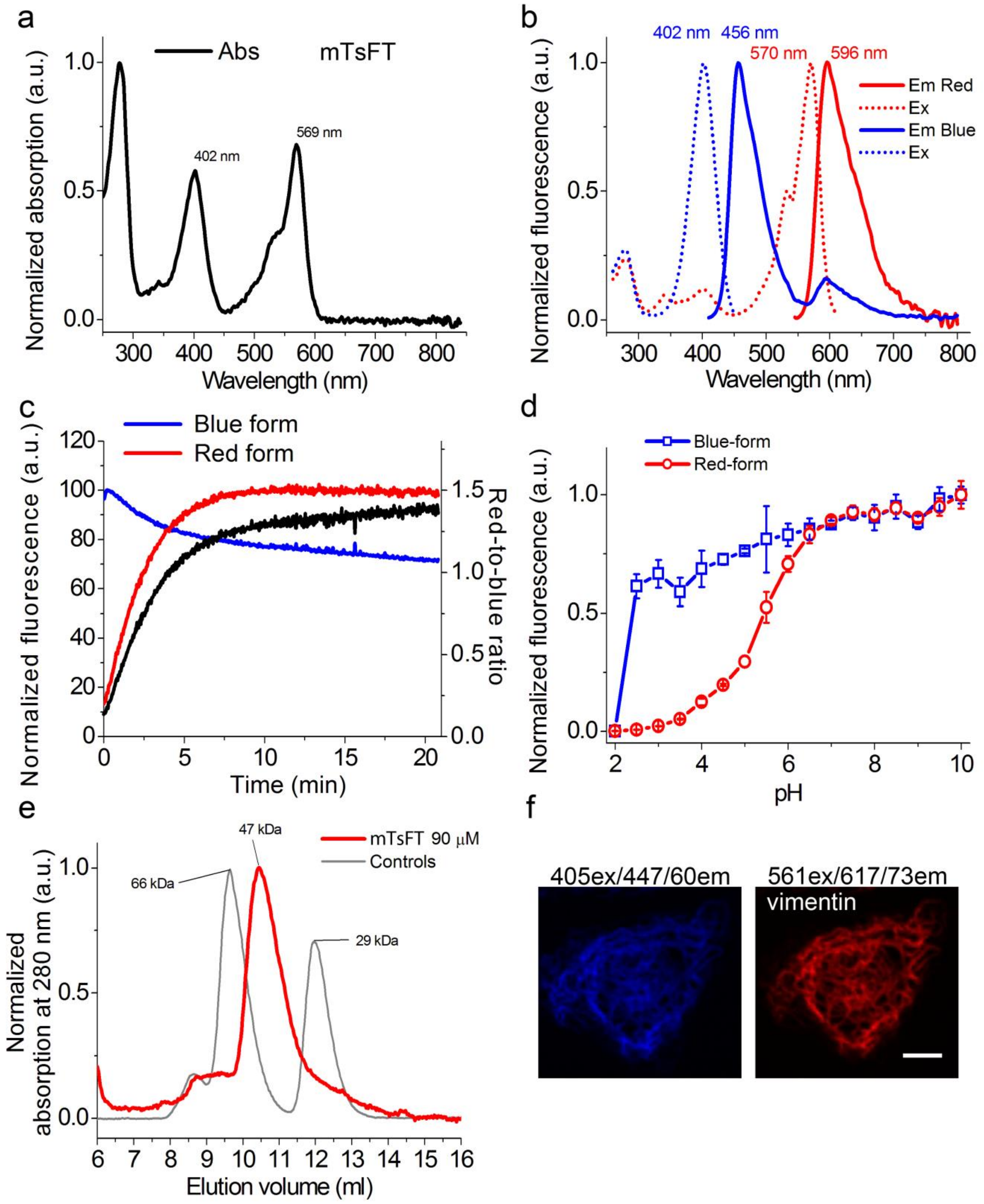

Figure S12. Properties of the purified mTsFT protein in vitro and in HeLa cells. (**a**) Absorption spectra for blue and red forms of mTsFT protein in PBS buffer at pH 7.40. (**b**) Excitation and emission spectra for blue and red forms of mTsFT in PBS buffer at pH 7.40. (**c**) Maturation of blue and red forms for mTsFT in PBS buffer at pH 7.40, 37 °C. Red-to-blue ratio was calculated according to the red and blue fluorescence time dependences normalized to 100. (**d**) Fluorescence intensity for blue and red forms of mTsFT as a function of pH. Three replicates were averaged for analysis. Error bars represent the standard deviation. (**e**) Fast protein liquid chromatography of mTsFT protein. Proteins were eluted in 20 mM Tris-HCl (pH 7.80) and 200 mM NaCl buffer. The molecular weight of mTsFT (57 kDa theoretical) was calculated from a linear regression of the dependence of logarithm of control molecular weights vs. elution volume. (**f**) Confocal images of HeLa Kyoto cells in blue (405ex and 447/60em) and red (561ex and 617/73em) channels 24 h after transfection with pVimentin-mTsFT plasmid. Scale bar: 10 μm.

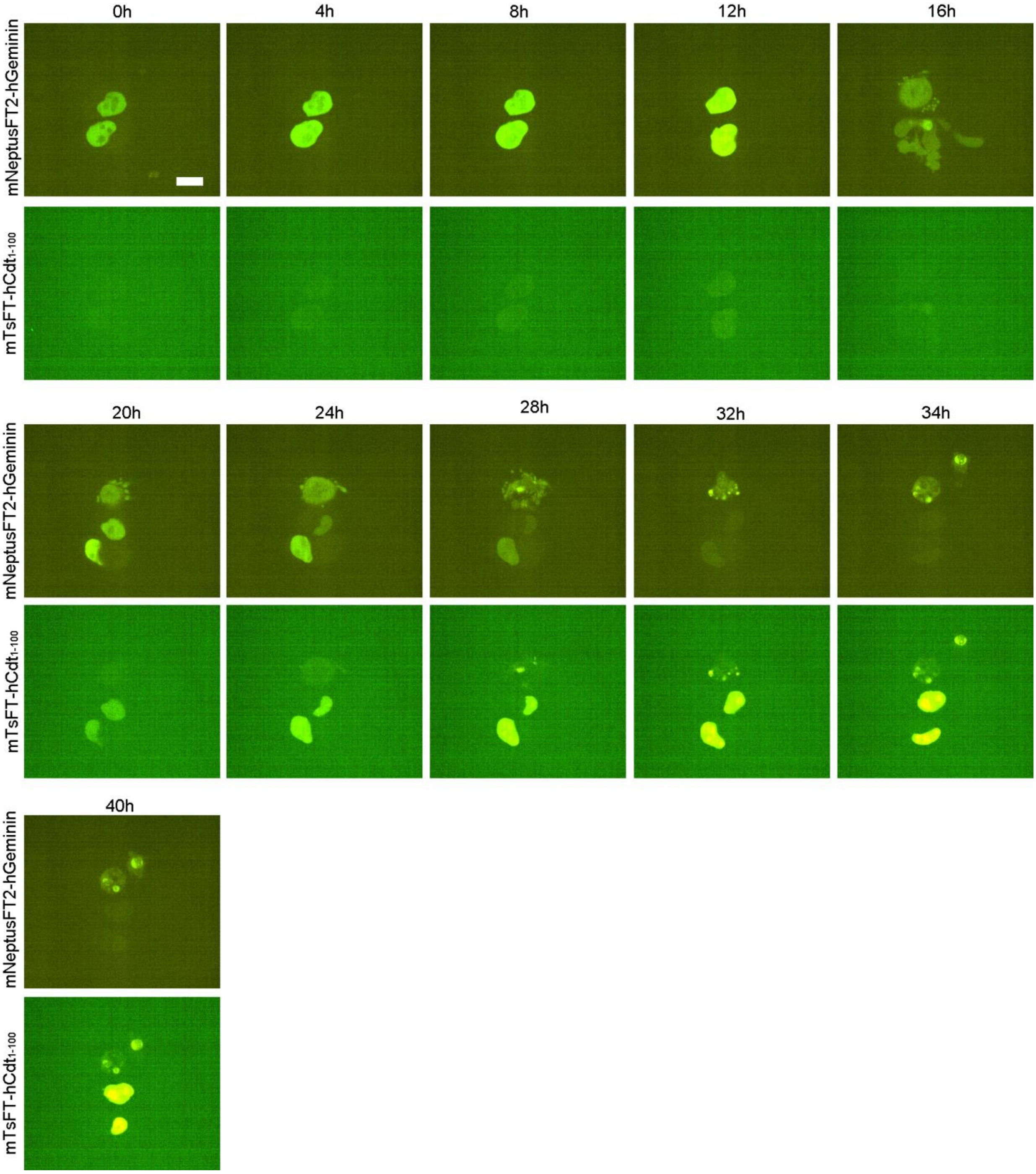

Figure S13. Visualization of the cell cycle using FucciFT1 system, which includes blue-to-red mTsFT-hCdt1-100 and green-to-far-red mNeptusFT2-hGeminin fusions. Confocal images of the HeLa cells are shown in green/far-red overlaid channels for mNeptusFT2-hGeminin fusion (in green and yellow pseudo colors, respectively) and in blue/red overlaid channels for mTsFT-hCdt1-100 fusion (in green and yellow pseudo colors, respectively). Scale bar: 15 μm.

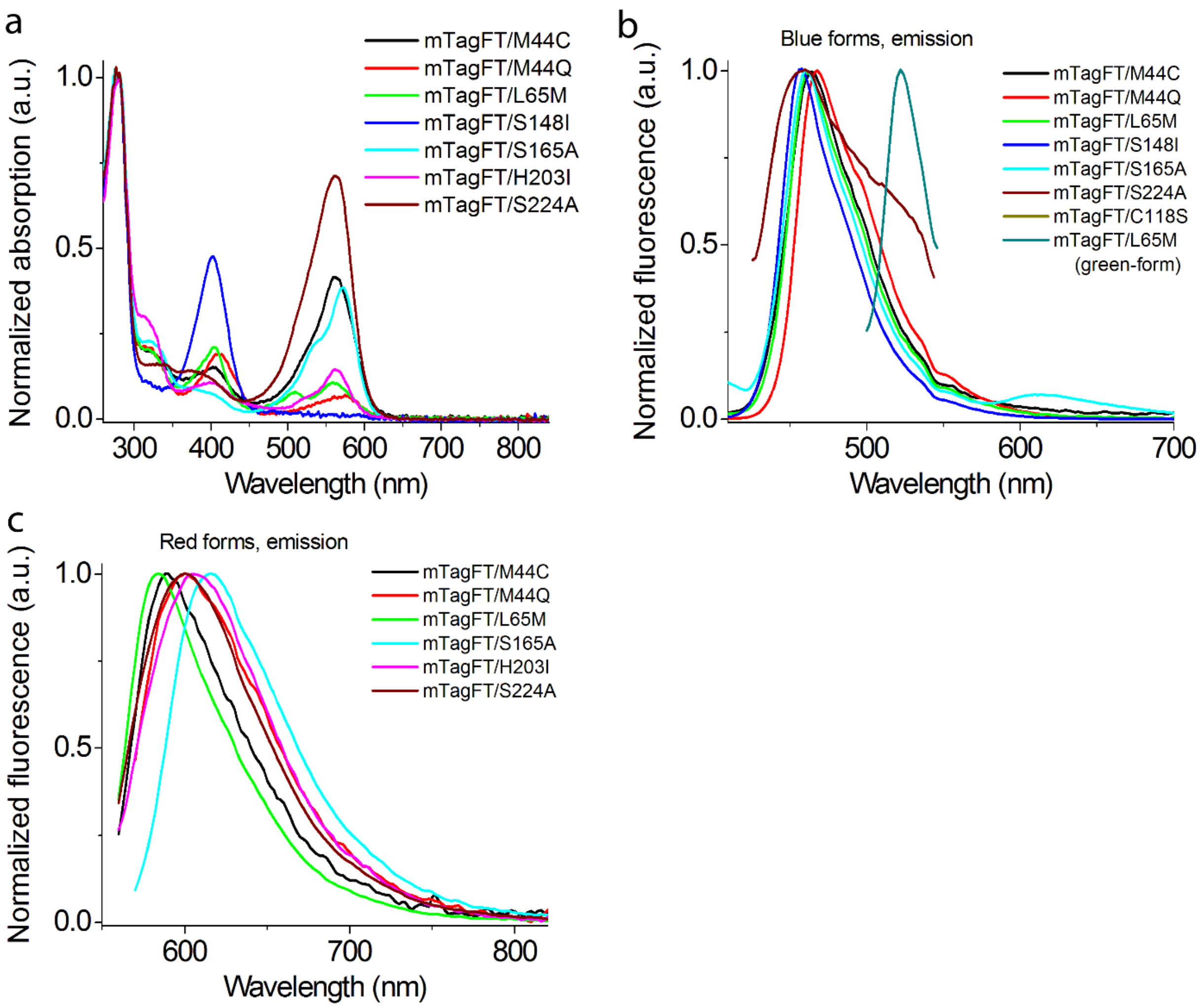

Figure S14. Spectral properties of the purified mTagFT mutants. Spectra were acquired in PBS buffer. For excitation of the blue and red forms we used 400 nm and 550-560 nm excitation light, respectively. Green form for mTagFT/L65M mutant was excited with 490 nm light.

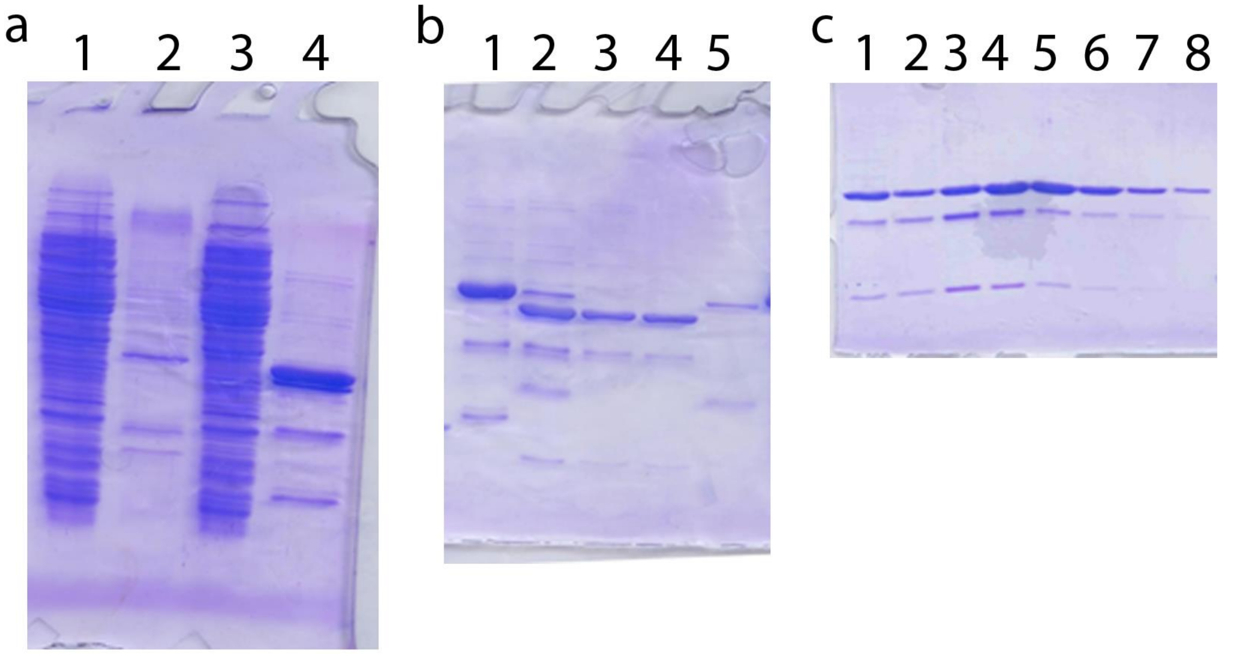

Figure S15. Analysis of the purification of the mTagFT protein for X-ray using electrophoresis in 15% PAGE. (**a**) Purification from lysate using Ni-NTA resin. Lanes: 1- lysate, 2- pellet, 3- slip from the column, 4- elution from the column. (**b**) Purification after proteolysis using Ni-NTA resin. Lanes: 1- protein before proteolysis, 2 – protein after proteolysis, 3, 4 – slip from the column after proteolysis, 5- elution from the column after proteolysis. (**c**) Purification using MonoS column. Lanes: 1- protein before purification, 2-8 elution fractions from the MonoS column.

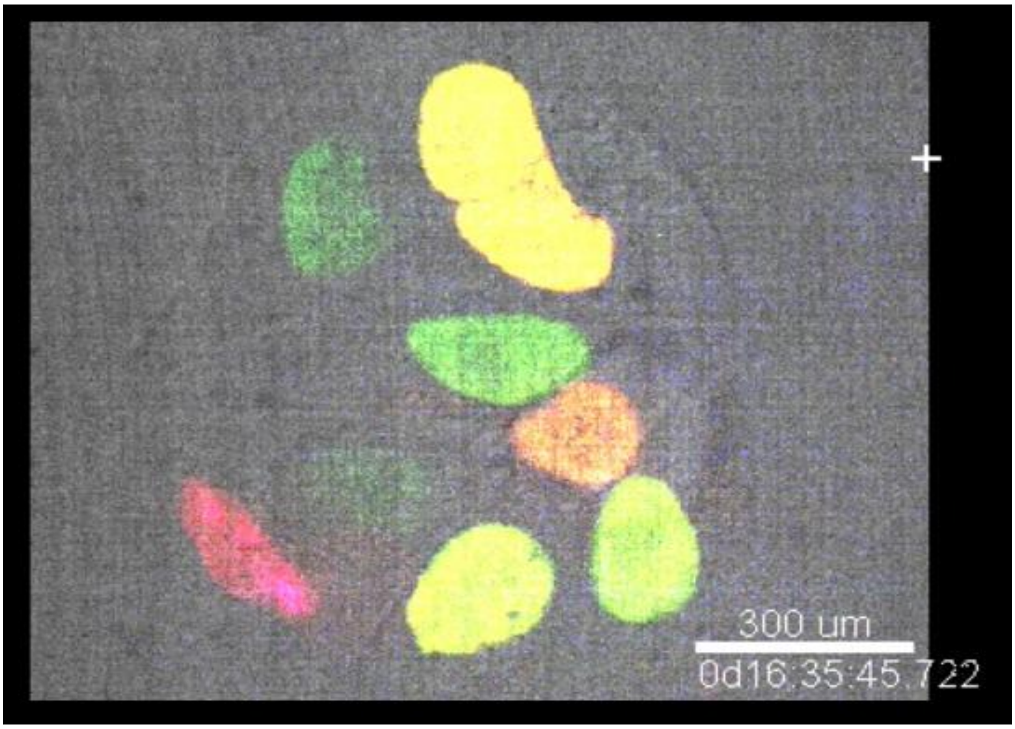

Video S1. Video describing work of the FucciFT2 system, which colors G1 and S/G2/M phases in blue/red and green/far-red (in yellow) colors, respectively. Confocal images of the HeLa cells stably expressing FucciFT2 system are shown in blue/green/red/far-red overlaid channels for blue-to-red TagFT-hCdt$_{1-100}$ (in blue and red overlaid pseudo colors, respectively) and green-to-far-red mNeptusFT2-hGeminin fusions (in green and yellow overlaid pseudo colors, respectively). Scale bar: 30 μm.

Supplementary Methods.

1. Cloning of Bacterial Plasmids, Mutagenesis and Library Screening

Timers and their variants were cloned into the pBAD/HisB plasmid (Invitrogen, Waltham, MA, USA) at the BglII/EcoRI or BglII/HindIII restriction sites using primers listed in Table S1 for expression of these proteins in BW25113 bacterial cells (kindly provided by Verkhusha V.V. from Albert Einstein College of Medicine, Bronx, NY, USA).

Random libraries for the development of timers were obtained using polymerase chain reaction (PCR) in the presence of Mn^{2+} ions in the conditions of 2–3 random mutations per 1000 base pairs (Diversify PCR Random Mutagenesis Kit User Manual, Clontech, Palo Alto, CA, USA) and cloned at the BglII/EcoRI restriction sites of the pBAD/HisB or pBAD/HisB-Hyper-sfGFP or pBAD/HisB-TorA-mTagBFP2-mScarlet plasmids. For PCR, the C1000 Touch Thermal Cycler (Bio-Rad, Hercules, California, USA) was used.

An overlap library for the rational mutagenesis of the parental TagRFP protein at positions 69, 152, 203, 205, and 224 was generated using primers listed in Table S1. The assembly of the whole genes was performed using PCR with overlapping fragments [1]. The generated library was inserted at the BglII/EcoRI restriction sites of the pBAD/HisB plasmid.

Directed mutagenesis of the mTagFT protein at positions 16, 44, 65, 148, 165, 203, 220 and 224 was performed using corresponding primers listed in Table S1. The assembly of the gene was performed using PCR with overlapping fragments [1]. The resulting genes were inserted at the BglII/EcoRI restriction sites into the pBAD/HisB plasmid.

The resulting PCR products were purified using a DNA purification kit (Eurogen, Russia). After the digestion of the pBAD/HisB plasmid and PCR products with the corresponding restriction enzymes, they were purified on a 1% agarose gel, followed by extraction using a DNA extraction gel kit (Eurogen, Moscow, Russia). After extraction, the plasmids and PCR products were ligated by T4 DNA ligase (3–16 h, at 16 °C), followed by purification of the ligated mixture with a DNA purification kit (Eurogen, Moscow, Russia). The purified ligation mixture was further transformed into electrocompetent bacteria BW25113 by electroporation using 1800 V pulse in an Eporate electroporator (Eppendorf, Hamburg, Germany) or chemically competent BW25113 bacterial cells.

The screening of the bacterial libraries was performed on Petri dishes under a fluorescent microscope. Briefly, the expression of the timers on the colonies on Petri dishes was induced with 0.2% arabinose at 37 °C. The screening of about 10,000 colonies of the bacterial library expressing FT variants was performed on Petri dishes under fluorescent stereomicroscope Leica M205FA (Leica, Wetzlar, Germany) equipped with the DFC310FX camera (Leica Microsystems, Wetzlar, Germany) and a mercury metal halide light source EL6000 (Leica Microsystems, Wetzlar, Germany). In case of TagFT, mTagFT and mTsFT timers, blue fluorescence was registered by 405/40BP excitation and 450/40BP emission filters; red fluorescence was registered by 540/40BP excitation and 620/60BP emission filters; we marked the bluest/non-red colonies 18 h after plating the bacteria on Petri dishes and selected the most red/non-blue colonies 72 h after plating. In case of mNeptusFT timers, green fluorescence was registered by 480/40BP excitation and 535/40BP emission filters; far-red fluorescence was registered by 620/60BP excitation and 700/75BP emission filters; we marked the most green/non-far-red colonies 18 h after plating the bacteria on Petri dishes and selected the most far-red/non-green colonies 72 h after plating. The acquired images were analyzed using ImageJ software, and the colonies with the largest contrast and brightness of both forms were selected for further analysis on bacterial streaks on Petri dishes, followed by protein purification and characterization.

After each round, 10–15 selected clones were purified and their properties characterized. To assess the green, red and far-red forms, the FT mutants were expressed in 10 mL of LB medium (10 g tryptone, 5 g yeast extract, and 10 g NaCl per 1L of water) containing ampicillin (100 µg/mL) and 0.002% arabinose for 16 h at 37 °C, 220 rpm and for 24 h at room temperature, followed by purification on Ni-NTA resin as described below. To characterize the blue form, the mutants were expressed in 100 mL of LB medium supplemented with ampicillin (100 µg/mL) in the absence of arabinose at 37 °C, 190 rpm, overnight. Then, 0.2% arabinose was added and the bacterial culture was incubated for 2–4 h at 37 °C, 190 rpm with a restriction of oxygen in 1L flasks closed with parafilm. The bacteria were precipitated by centrifugation for 12 min at 3500 rpm. The proteins were further extracted from the pellet with 300 µL of B-Per extraction reagent containing lysozyme (1 mg/mL final concentration) and DNase I (1 unit/µL) by shaking the pellet for 20 min at 37 °C and 200 rpm. Alternatively, bacterial cells were resuspended in 10

ml of PBS supplemented with 100 mM NaCl and 10 mM Imidazole and sonicated on ice (for 2 min, in the cycle of 30 sec pulse, and 30 sec pause; 20% power of the VCX130 Sonicator and CV18 tip (Sonics & Materials Inc., Newtown, CT, USA). Next, the lysed components of the bacteria were removed by centrifugation for 2 min at 13,200 rpm. The protein supernatant was further bound to 50-150 μL of Ni-NTA resin for 30–60 min on an orbital shaker at 4 °C. Next, the resin with bound protein was washed twice with 1 mL of a PBS buffer supplemented with 100 mM NaCl and 10 mM Imidazole. The protein was then eluted from a column packed with resin with the PBS buffer supplemented with 100 mM NaCl and 400 mM imidazole. Finally, using a spectrofluorometer and spectrophotometer, the characteristic spectral properties and brightness were determined for the purified proteins, as described in Section 3.2.

2. Proteins' Purification and Characterization

For the final characterization, the proteins were expressed and purified using the pBAD/HisB arabinose-inducible system (Invitrogen, Waltham, MA, USA) from 400 mL of medium, as described in Section 3.1 with modifications. Briefly, for red-form protein expression, the bacterial cultures were grown in 400 mL of LB medium supplemented with 0.004% arabinose and 100 μg/mL ampicillin overnight at 37 °C and 220 rpm. For blue-form protein expression, the bacterial cultures were grown in 400 mL of LB medium supplemented with 100 μg/mL ampicillin overnight at 37 °C and 220 rpm; the next day, the protein expression was induced with 0.2% arabinose for 4 h at 37 °C and 220 rpm. The cultures were then centrifuged at 4648× *g* for 10 min. The cell pellets were resuspended in PBS buffer, pH 7.4, supplemented with 300 mM NaCl (buffer A) and 10 mM imidazole, and lysed by sonication on ice (for 8 min, in the cycle of 30 sec pulse, and 30 sec pause; 20% power of the VCX130 Sonicator and CV18 tip (Sonics & Materials Inc., Newtown, CT, USA). The sonicated solution was centrifuged at 36,670× *g* at 4 °C for 4 min. The proteins were further bound with 1–1.5 mL of Ni-NTA resin (Qiagen, Germantown, MD, USA) for 30 min on ice with mixing. Resin with bound protein was twice washed with buffer A. The proteins were eluted with 400 mM imidazole in buffer A. The collected fractions with eluted protein of 1–1.5 mL were dialyzed against PBS buffer for 16 h.

The extinction coefficient values for the blue form of purified TagFT and mTagFT proteins and their derivatives were calculated in PBS buffer, pH 7.4, using the acid denaturation method and assuming that the TagBFP-like chromophore has the extinction coefficient of 28,500 M^{-1} cm^{-1} at 382 nm in 1M HCl [2]. The extinction coefficient values for the red form of purified TagFT and mTagFT proteins were calculated in PBS buffer, pH 7.4, relative to the absorption peak at 280 nm, assuming the extinction coefficient at 280 nm of 26,025 and 39,880 M^{-1} cm^{-1}, respectively. The extinction coefficients of the far-red form of mNeptusFT1 and mNeptusFT2 protein were calculated relative to the sfGFP absorption at 490 nm assuming extinction coefficient of 83300 $M^{-1}cm^{-1}$ [3]. The extinction coefficients of the blue form of mTsFT protein was calculated relative to the mScarlet absorption at 569 nm assuming extinction coefficient of 100000 $M^{-1}cm^{-1}$ [4]. The absorption spectra were recorded using a NanoDrop 2000c Spectrophotometer (Thermo Scientific, Waltham, MA, USA).

The quantum yields for the blue forms of the purified TagFT, mTagFT and mTsFT proteins and their derivatives excited at 400 nm were measured by a comparison of the integrated fluorescence values (in the range of 410–800 nm) in PBS buffer, pH 7.40, with the similarly integrated fluorescence values for the equally absorbing at 400 nm mTagBFP2 protein (quantum yield of 0.64 [5]). The quantum yields for the red form of the purified TagFT and mTagFT proteins and their derivatives excited at 540 nm were measured by a comparison of the integrated fluorescence values (in the range of 550–820 nm) in PBS buffer, pH 7.40, with the similarly integrated fluorescence values for the equally absorbing at 540 nm mCherry protein (quantum yield of 0.22 [6]). The quantum yields for the far-red form of the purified mNeptusFT1/2 proteins excited at 590 nm were measured by a comparison of the integrated fluorescence values (in the range of 600–820 nm) in PBS buffer, pH 7.40, with the similarly integrated fluorescence values for the equally absorbing at 590 nm smURFP protein with QY of 0.18 [7]. The fluorescence spectra were acquired using a CM2203 spectrofluorometer (SOLAR, Minsk, Belarus).

The pH titrations for the purified proteins (1.2 μM final concentration) were performed in a buffer of 30 mM citric acid, 30 mM borax, and 30 mM NaCl with a pH adjusted from 3.0 to 10.5, after incubation for 20 min at room temperature. Blue (Ex 365 nm/Em 410–460 nm), green (Ex 490/Em 510-570 nm), red (Ex 525 nm/Em 580–640 nm) and far-red (Ex 625 nm/Em 660-720 nm) fluorescence were registered using a 96-well Modulus™ II Microplate Reader (Turner Biosystems, Sunnyvale, CA, USA).

Size-exclusion chromatography was performed with a Superdex™ 75 10/300 GL column using the GE AKTA Explorer 100 (Amersham Pharmacia, UK) FPLC System.

To assess the maturation rate of timers, 100 mL of bacterial cultures were grown in a 1 L flask with LB medium supplemented with 100 µg/mL ampicillin at 37 °C, 190 rpm, overnight. Next, protein expression was induced by the addition of 0.2% arabinose, and the flask throat was closed using parafilm. The protein expression lasted for 2–4 h at 37 °C, 190 rpm, under anaerobic conditions. The cultures were then centrifuged at 3500× *g* for 12 min at room temperature. The protein was purified on ice using Ni-NTA resin. A total of 100 µL of purified protein was mixed with 2.9 mL of PBS buffer supplemented (pre-warmed at 37 °C for 10 min) in a 5 mL quartz cuvette. Fluorescence kinetics were further measured using the CM2203 spectrofluorometer (SOLAR, Minsk, Belarus) at 37 °C with registration of both blue (Ex 400 nm/Em 460 nm) and red fluorescence (Ex 580 nm/Em 630 nm) changes over time.

Alternatively, to determine the maturation rate of timers, 20 ml of bacterial cultures were grown in a 50 mL tubes with LB medium supplemented with 100 µg/mL ampicillin at 37 °C, 190 rpm, overnight. Next day, protein expression was induced by the addition of 0.2% arabinose (200 mkl of 20% arabinose), and the culture was transferred into 15 ml tube with closed lid and filled till the brim. The protein expression lasted for 3 h at 37 °C, 190 rpm, under anaerobic conditions. The cultures were then centrifuged at 3500× *g* for 10 min at room temperature. The bacterial pellet was resuspended in 800 mkl of PBS buffer supplemented with chloramphenicol antibiotics and sonicated in 2 ml tube for 40 sec using 20% power of the VCX130 Sonicator and CV18 tip (Sonics & Materials Inc., Newtown, CT, USA). Lysate was centrifuged at 46090 g for 4 min at 0°C. A total of 100 µL of purified protein was mixed with 2.9 mL of PBS buffer supplemented with chloramphenicol (pre-warmed at 37 °C for 10 min) in a 5 mL quartz cuvette. Fluorescence kinetics were further measured using the CM2203 spectrofluorometer (SOLAR, Minsk, Belarus) at 37 °C with registration of both blue (Ex 400 nm/Em 460 nm) and red fluorescence (Ex 580 nm/Em 630 nm) each 10 sec for 1200 min and more time and using high sensitivity, average time of 2.0 sec and slits 4.0 nm settings.

Photobleaching experiments were performed with suspensions of purified proteins in mineral oil, as previously described [8]. Briefly, the kinetics of photobleaching were measured using purified proteins dialyzed in PBS buffer at a 45 µM concentration in aqueous microdroplets in mineral oil using a Zeiss Axio Imager Z2 microscope (Zeiss, Germany) equipped with a 120 W mercury short-arc lamp (LEJ, Germany), a 63 × 1.4 NA oil immersion objective lens (PlanApo, Zeiss, Germany), a 550/25BP excitation filter, a FT 570 beam splitter, and 605/70BP emission filters. Light power density (9.136 mW/cm^2) was measured at the rear focal plane of the objective lens using a PM100D power meter (ThorLabs, Germany) equipped with an S120VS sensor (ThorLabs, Germany). No corrections were applied to the experimental data.

3. Purification of the mTagFT protein for crystallization.

For the preparative purification of the mTagFT protein for X-ray crystallography, bacterial cells expressing the mTagFT protein with N-terminal His-tag and the Tobacco Etch Virus (TEV) protease cleavage site were pelleted by centrifugation for 20 min at 5000 rpm and 4 °C (Beckman Coulter centrifuge, Brea, CA, USA). Then, the pellet (pellet weight was 15.1 g from 2.6 L of medium) was resuspended in 100 mL of buffer A (40 mM Tris-HCl, pH 7.8, containing 400 mM NaCl and 10 mM imidazole) supplemented with 0.2% Triton X-100, and 1 mM phenylmethylsulfonyl fluoride, and disrupted by ultrasound sonication (2 sec pulse, 6 sec pause, amplitude 45%, total time 5 min). The crude cell extract was centrifuged for 30 min at 28,000× *g* and 4 °C (Beckman Coulter centrifuge, Brea, CA, USA). The supernatant was loaded onto a 5 mL Ni-NTA Superflow column (Qiagen, Hilden, Germany) equilibrated with buffer A supplemented with 0.1% (*v*/*v*) Triton X-100. Then, there were sequential washes with buffer A and buffer A supplemented with 40 mM imidazole. Protein elution was performed with buffer A supplemented with 300 mM imidazole (Figure S12a). Amounts of 1 mM DTT and 1 mM EDTA were added to the protein solution, mixed with TEV protease (1 mg per 10 mg protein), and the whole mixture was dialyzed for 16 h in buffer B (40 mM Tris, pH 7.8, 400 mM NaCl, 5 mM imidazole, 2 mM BME, 1 mM EDTA) at +4 °C; His-tag cleavage was controlled by electrophoresis in polyacrylamide gel (PAGE), gel concentration 15% (Figure S6b). The digested protein was then loaded onto a Ni-NTA Superflow column (Qiagen, EU) equilibrated with buffer B; TEV protease and cleaved His-tag bound to a Ni-NTA Superflow column (Qiagen, EU), and the leaked protein was concentrated to 1.0 mL using a 10 kDa cut-off concentrator (Millipore, Burlington, MA, USA) and loaded onto a HiTrap Desalting column (GE Healthcare, Danderyd, Sweden) in buffer 50 mM Na Phosphate buffer. For further

purification, protein was loaded onto the MonoS (GE Healthcare, Danderyd, Sweden) column equilibrated with 50 mM phosphate buffer pH 7.0. Elution of protein was performed using linear gradient of NaCl concentration, protein eluted at 120-150 mM NaCl (Figure S12c). The fractions containing the target protein were concentrated to 1.2 mL using 10 kDa cut-off concentrators (Millipore, Burlington, MA, USA) and transferred into 20 mM Tris buffer pH 8.0, 150 mM NaCl on a PD-10 column (GE Healthcare, Danderyd, Sweden). Then, the protein concentration was measured by the bicinchoninic method using the Bicinchoninic Acid Protein Assay Kit (Sigma-Aldrich, Saint Louis, MO, USA). BSA protein standard P0914-5AMP solution (Sigma-Aldrich, Saint Louis, MO, USA) was used as a standard. A 0.5 ml solution of 3.5 mg/mL was obtained. The total protein yield was 1.75 mg. The purity of the preparations at all stages was monitored by electrophoresis in PAGE (gel concentration 15%). Protein chromatography was performed using the ÄKTA prime plus and ÄKTA explorer 100 systems (GE Healthcare, Danderyd, Sweden).

4. Data Collection, Processing, Structure Solution, and Refinement

mTagFT crystals were briefly soaked in a crystallization solution supplemented with PEG 400 25%(Hampton Research, Aliso Viejo, CA, USA) immediately prior to diffraction data collection and flash-frozen in liquid nitrogen. The crystals were preliminarily tested for their diffraction quality at the beamline "Belok-RSA" of the Kurchatov SNC (Moscow, Russia) [9]. The 2.9Å data were finally collected at 100K at BL41XU beamline (SPring8, Sayo, Hyogo, Japan). The data were indexed, integrated, and scaled using the DIALS program [10] (Table 4).

The structure was solved by the molecular replacement method using the MOLREP program [11] and the structure of the fluorescent protein TagRFP (PDB ID: 3M22) as an initial model. The refinement of the structure was carried out using the REFMAC5 [12] and BUSTER [13] programs of the CCP4 suite [14] using TWIN option. The visual inspection of electron density maps and the manual rebuilding of the model were carried out using the COOT interactive graphics program [15]. The hydrogen atoms in fixed positions, as well as TLS and NCS options. In the final model, an asymmetric unit contained four copies of the protein of 220 residues with the partially modelled chromophore and 158 water molecules. The first seven residues from the N-terminal as well as the last ten residues from the C-terminal part of the protein were not visible in the electron density due to disorder.

5. Structure Analysis and Validation

The visual inspection of the structure was carried out using the COOT program and the PyMOL Molecular Graphics System, Version 1.9.0.0 (Schrödinger, New York, NY, USA). The structure comparison and superposition were made using the PDBeFold program [16], while contacts were analyzed using the PDBePISA [17].

6. Mammalian Plasmids Construction

In order to construct the pAAV-*CAG*-mTagFT-P2A-EGFP, pAAV-*CAG*-TagFT-P2A-EGFP, pAAV-*CAG*-mTsFT-P2A-EGFP, plasmids, the mTagFT, TagFT and mTsFT genes were PCR amplified as the KpnI-AgeI fragments, using TagFT-NheI2/TagFT-AgeI-r or Neptune-KpnI/RubyFT-AgeI-r primers listed in Table S1, and swapped with the iRFP gene in the pAAV-*CAG*-iRFP-P2A-EGFP vector.

In order to construct the pmTagFT-actin plasmid, the mTagFT gene was PCR amplified as the NheI-BglII fragment, using TagFT-NheI2/TagFT-BglII-r primers listed in Table S2, and swapped with the TagBFP gene in the pTagBFP-actin vector (Evrogen, Moscow, Russia).

In order to construct the pmTagFT-tubulin plasmid, the mTagFT gene was PCR amplified as the NheI-BglII fragment, using TagFT-NheI2/TagFT-BglII-r primers listed in Table S2, and swapped with the TagGFP2 gene in the pTagGFP2-tubulin vector (Evrogen, Moscow, Russia).

In order to construct the pLU-vimentin-mTagFT and pLU-vimentin-TagFT plasmids, the mTagFT and TagFT genes were PCR amplified as the BamHI-BsrGI and AgeI/XbaI fragments, using BamHI-mTFT/mTFT-XbaI-r primers listed in Table S2, and swapped with the NeonOxIrr gene in the pLU-vimentin-NeonOxIrr vector [18].

In order to construct the pLU-vimentin-mTsFT and pLU-vimentin-mNeptusFT2 plasmids, the mTsFT and mNeptusFT2 genes were PCR amplified as the AgeI-XbaI fragments, using BamHI-Neptune/LSSmSc-XbaI-r primers listed in Table S2, and swapped with the TagFT gene in the pLU-vimentin-TagFT vector.

In order to construct the pSBbi-GN-mNeptusFT1/2-hGeminin(1-110)-Puromycin, pSBbi-GN-mTsFT-hCdt1(1-100)-Hygromycin, pSBbi-GN-MediumFT-hCdt1(1-100)-Hygromycin, pSBbi-GN-TagFT-hCdt1(1-100)-Hygromycin, pSBbi-GN-FastFT-hCdt1(1-100)-Hygromycin, pSBbi-GN-FastFT2-hCdt1(1-100)-Hygromycin plasmids, the mNeptusFT1/2-hGeminin(1-110), mTsFT-hCdt1(1-100), MediumFT-hCdt1(1-100), TagFT-hCdt1(1-100), FastFT-hCdt1(1-100), and FastFT2-hCdt1(1-100) genes were PCR amplified as the KpnI-HindIII fragments, using Neptune-KpnI/hGem-HindIII-r and FT-KpnI/hCdt1-HindIII-r primers listed in Table S2, and swapped with the iRFP gene in the pSBbi-GN-iRFP-hCdt1(1-100)-Puromycin and pSBbi-GN-iRFP-hCdt1(1-100)-Hygromycin vectors derived from pSBbi-GN vector (Addgene #60517).

7. Mammalian Live Cell Imaging

Transient transfection of the HeLa Kyoto cells was performed in a 24-well format using lipofectamine reagent according to the manufacturer’s protocol. Cells were cultured using DMEM medium supplemented with 10% FBS, glutamine, 50 U/mL penicillin, and 50 U/mL streptomycin, at 37 °C and 5% CO_2. HeLa cell cultures were imaged 24–72 h after the transient transfection using a laser spinning-disk Andor XDi Technology Revolution multi-point confocal system (Andor Technology, Belfast, UK) equipped with an inverted Nikon Eclipse Ti-E/B microscope (Nikon Instruments, Tokyo, Japan), a 75 W mercury–xenon lamp (Hamamatsu, Hamamatsu, Japan), a 60× oil immersion objective NA 1.4 (Nikon, Tokyo, Japan), a 16-bit Neo sCMOS camera (Andor Technology, Belfast, UK), a laser module Revolution 600 (Andor Technology, Belfast, UK), and a spinning-disk module Yokogawa CSU-W1 (Andor Technology, Belfast, UK). The blue, green, red and far-red fluorescence were acquired using the 405, 488, 561, 640 nm lasers, a confocal dichroic mirror 405/488/561/640, and filter wheel emission filters 447/60, 525/50, 617/73, and 685/40 nm, respectively. During imaging, the cells were incubated at 37 °C and 5% CO_2 using a cage incubator (Okolab, Naples, Italy).

References

1. Ho, S.N.; Hunt, H.D.; Horton, R.M.; Pullen, J.K.; Pease, L.R. Site-directed mutagenesis by overlap extension using the polymerase chain reaction. *Gene* **1989**, *77*, 51–59.
2. Subach, O.M.; Gundorov, I.S.; Yoshimura, M.; Subach, F.V.; Zhang, J.; Gruenwald, D.; Souslova, E.A.; Chudakov, D.M.; Verkhusha, V.V. Conversion of red fluorescent protein into a bright blue probe. *Chem. Biol.* **2008**, *15*, 1116–1124.
3. Pedelacq, J.D.; Cabantous, S.; Tran, T.; Terwilliger, T.C.; Waldo, G.S. Engineering and characterization of a superfolder green fluorescent protein. *Nat. Biotechnol.* **2006**, *24*, 79–88.
4. Bindels, D.S.; Haarbosch, L.; van Weeren, L.; Postma, M.; Wiese, K.E.; Mastop, M.; Aumonier, S.; Gotthard, G.; Royant, A.; Hink, M.A.; et al. mScarlet: A bright monomeric red fluorescent protein for cellular imaging. *Nat. Methods* **2017**, *14*, 53–56.
5. Subach, O.M.; Cranfill, P.J.; Davidson, M.W.; Verkhusha, V.V. An enhanced monomeric blue fluorescent protein with the high chemical stability of the chromophore. *PLoS ONE* **2011**, *6*, e28674.
6. Shaner, N.C.; Campbell, R.E.; Steinbach, P.A.; Giepmans, B.N.; Palmer, A.E.; Tsien, R.Y. Improved monomeric red, orange and yellow fluorescent proteins derived from Discosoma sp. red fluorescent protein. *Nat. Biotechnol.* **2004**, *22*, 1567–1572.
7. Rodriguez, E.A.; Tran, G.N.; Gross, L.A.; Crisp, J.L.; Shu, X.; Lin, J.Y.; Tsien, R.Y. A far-red fluorescent protein evolved from a cyanobacterial phycobiliprotein. *Nat. Methods* **2016**, *13*, 763–769.
8. Doronin, D.A.; Barykina, N.V.; Subach, O.M.; Sotskov, V.P.; Plusnin, V.V.; Ivleva, O.A.; Isaakova, E.A.; Varizhuk, A.M.; Pozmogova, G.E.; Malyshev, A.Y.; et al. Genetically encoded calcium indicator with NTnC-like design and enhanced fluorescence contrast and kinetics. *BMC Biotechnol.* **2018**, *18*, 10.
9. Svetogorov, R.; Dorovatovskii, P.; Lazarenko, V. Belok/XSA Diffraction Beamline for Studying Crystalline Samples at Kurchatov Synchrotron Radiation Source. *Cryst. Res. Technol.* **2020,** 55, 1900184.
10. Winter, G.; Waterman, D.G.; Parkhurst, J.M.; Brewster, A.S.; Gildea, R.J.; Gerstel, M.; Fuentes-Montero, L.; Vollmar, M.; Michels-Clark, T.; Young, I.D.; et al. DIALS: Implementation and evaluation of a new integration package. *Acta Crystallogr. D Struct. Biol.* **2018,** 74, 85-97.
11. Vagin, A.; Teplyakov, A. MOLREP: An automated program for molecular replacement. *J. Appl. Crystallogr.* **1997**, *30*, 1022–1025.
12. Murshudov, G.N.; Vagin, A.A.; Lebedev, A.; Wilson, K.S.; Dodson, E.J. Efficient anisotropic refinement of macromolecular structures using FFT. *Acta Crystallogr. Sect. D Biol. Crystallogr.* **1999,** 55, 247–255.
13. Bricogne, G.; Blanc, E.; Brandl, M.; Flensburg, C.; Keller, P.; Paciorek, W.; Roversi, P.; Sharff, A.; Smart, O.S.; Vonrhein, C.; BUSTER version 2.10.4. Global Phasing Ltd.: Cambridge, UK, 2011.
14. The CCP4 suite: Programs for protein crystallography. *Acta Crystallogr D Biol Crystallogr* **1994,** 50, 760–763.
15. Emsley, P.; Cowtan, K. Coot: Model-building tools for molecular graphics. *Acta Crystallogr D Biol Crystallogr* **2004,** 60, 2126–2132.
16. Krissinel, E.; Henrick, K. Secondary-structure matching (SSM), a new tool for fast protein structure alignment in three dimensions. *Acta Crystallogr. D Biol. Crystallogr.* **2004,** 60, 2256–2268.
17. Krissinel, E.; Henrick, K. Inference of macromolecular assemblies from crystalline state. *J. Mol. Biol.* **2007**, *372*, 774–797.
18. Subach, O.M.; Kunitsyna, T.A.; Mineyeva, O.A.; Lazutkin, A.A.; Bezryadnov, D.V.; Barykina, N.V.; Piatkevich, K.D.; Ermakova, Y.G.; Bilan, D.S.; Belousov, V.V.; et al. Slowly Reducible Genetically Encoded Green Fluorescent Indicator for In Vivo and Ex Vivo Visualization of Hydrogen Peroxide. *Int. J. Mol. Sci.* **2019**, *20*, 3138.
